# The role of vasculature and angiogenesis in respiratory diseases

**DOI:** 10.1007/s10456-024-09910-2

**Published:** 2024-04-05

**Authors:** Maximilian Ackermann, Christopher Werlein, Edith Plucinski, Sophie Leypold, Mark P. Kühnel, Stijn E. Verleden, Hassan A. Khalil, Florian Länger, Tobias Welte, Steven J. Mentzer, Danny D. Jonigk

**Affiliations:** 1 Institute of Pathology, University Clinics of RWTH University, Aachen, Germany; 2 Institute of Pathology and Molecular Pathology, Helios University Clinic Wuppertal, University of Witten/Herdecke, Witten, Germany; 3 Institute of Anatomy, University Medical Center of the Johannes Gutenberg-University, Mainz, Germany; 4 Institute of Pathology, Hannover Medical School, Hannover, Germany; 5 Member of the German Center for Lung Research (DZL), Biomedical Research in Endstage and Obstructive Lung Disease Hannover (BREATH), Hannover, Germany; 6 Antwerp Surgical Training, Anatomy and Research Centre (ASTARC), University of Antwerp, Antwerp, Belgium; 7 Division of Thoracic and Cardiac Surgery, Department of Surgery, Brigham and Women’s Hospital, Boston, USA; 8 Laboratory of Adaptive and Regenerative Biology, Brigham & Women’s Hospital, Harvard Medical School, Boston, MA USA; 9 Department of Respiratory Medicine, Hannover Medical School, Hannover, Germany

**Keywords:** Respiratory diseases, Intussusceptive angiogenesis, Fibrovascular interface, Vascular normalization, Endothelial mesenchymal transition (EndoMT), Tumor angiogenesis, ECFC, COVID-19, Pulmonary fibrosis, COPD

## Abstract

In European countries, nearly 10% of all hospital admissions are related to respiratory diseases, mainly chronic life-threatening diseases such as COPD, pulmonary hypertension, IPF or lung cancer. The contribution of blood vessels and angiogenesis to lung regeneration, remodeling and disease progression has been increasingly appreciated. The vascular supply of the lung shows the peculiarity of dual perfusion of the pulmonary circulation (vasa publica), which maintains a functional blood-gas barrier, and the bronchial circulation (vasa privata), which reveals a profiled capacity for angiogenesis (namely intussusceptive and sprouting angiogenesis) and alveolar-vascular remodeling by the recruitment of endothelial precursor cells. The aim of this review is to outline the importance of vascular remodeling and angiogenesis in a variety of non-neoplastic and neoplastic acute and chronic respiratory diseases such as lung infection, COPD, lung fibrosis, pulmonary hypertension and lung cancer.

## Introduction

According to the WHO, lung diseases are the second leading cause of death worldwide. These include in particular destructive chronic lung diseases such as chronic obstructive pulmonary disease (COPD), lung cancer or pulmonary fibrosis [1]. The pathogenesis of all these diseases is shared by the occurrence of significant regenerative remodeling processes or compensatory lung growth, in particular of the pulmonary vascularity which spans a capillary network of more than 3000 km and makes up to more than 40% of all lungs cells [2]. While compensatory lung growth was observed in numerous animals after pneumonectomy [3, 4] recent clinical evidence suggests that compensatory lung growth and neoalveolarization continues throughout childhood and adolescence in humans [5]. Although the impact of stem cell lineages on this lung growth and regeneration has been intensively studied, there is limited understanding about the pulmonary vascularity during these compartment-specific regenerative processes [6, 7]. Thereby, the preservation of an intact vascular architecture is considered crucial to maintain the functional pulmonary blood-gas-barrier. The development and remodeling of the pulmonary vasculature is a complex morphogenetic process that requires not only the formation of new vessels, but also the successful matching of ventilation and perfusion. Although the pulmonary vasculature shares unique characteristics of dual sources of perfusion, namely pulmonary and bronchial circulation, bronchial vessels are considered as the main conductor for lung remodeling or tumor angiogenesis ([8–10]. Bronchial vessels originating from the aorta primarily supply the vessels of the submucosa of the bronchus and the bronchial walls. Morphological evidence showed the presence of numerous arteriovenous anastomoses ([11, 12] and “Sperrarterien” [12] between pulmonary and bronchial circulation which are involved in the regulation of intrapulmonary arteriovenous shunting. The “Sperrarterien” are found in the subpleural tissue, mainly at the septal margin of secondary pulmonary lobules as well as on the mediastinal pulmonary surface. The pulmonary circulation accounts for 97% of total circulation in the lung and the bronchial circulation for 3% under physiological conditions [13] where the intrapulmonary perfusion by bronchial vessels, bronchopulmonary anastomoses and “Sperrarterien” dramatically increased in severe hypoxic lung conditions as e.g. in pulmonary hypertension [14], asthma [15] or COVID-19 [10, 16].

However, not only does the blood supply by pulmonary and bronchial circulation show different patterns, but there also differences in the dynamics and plasticity of pulmonary endothelial cells. In this regard, pulmonary endothelial cells are distinct from the systemic vascular bed: while they are exposed to the highest oxygen tension and a low-pressure blood flow [17] this, however, facilitates inflammation, coagulation, and the interaction with blood-borne cells. In general, pulmonary-venous lung capillaries are continuous, non-fenestrated vessels with a lacking expression of Weibel-Palade bodies, endothelial storage granules containing von Willebrand factor and p-selectin, on the alveolar capillary level [18, 19], whereas systemically perfused vessels of bronchi and the pleural spaces are characterized by a fenestrated endothelium with Weibel-Palade bodies and by the evidence for distinct pro-angiogenic remodeling and permeability [20, 21]. Thereby, lung remodeling and growth is predominantly characterized by the recruitment and migration of blood-borne monocytes, CD34 + -circulating angiogenic progenitor cells, ATII cells, and resident and migratory myofibroblasts [22–24] and epithelial- or mesothelial-mesenchymal-transition in the subpleural or interlobular regions which are mainly supplied by the bronchial circulation [10].

## Mechanisms of angiogenesis

**Fig. 1:**
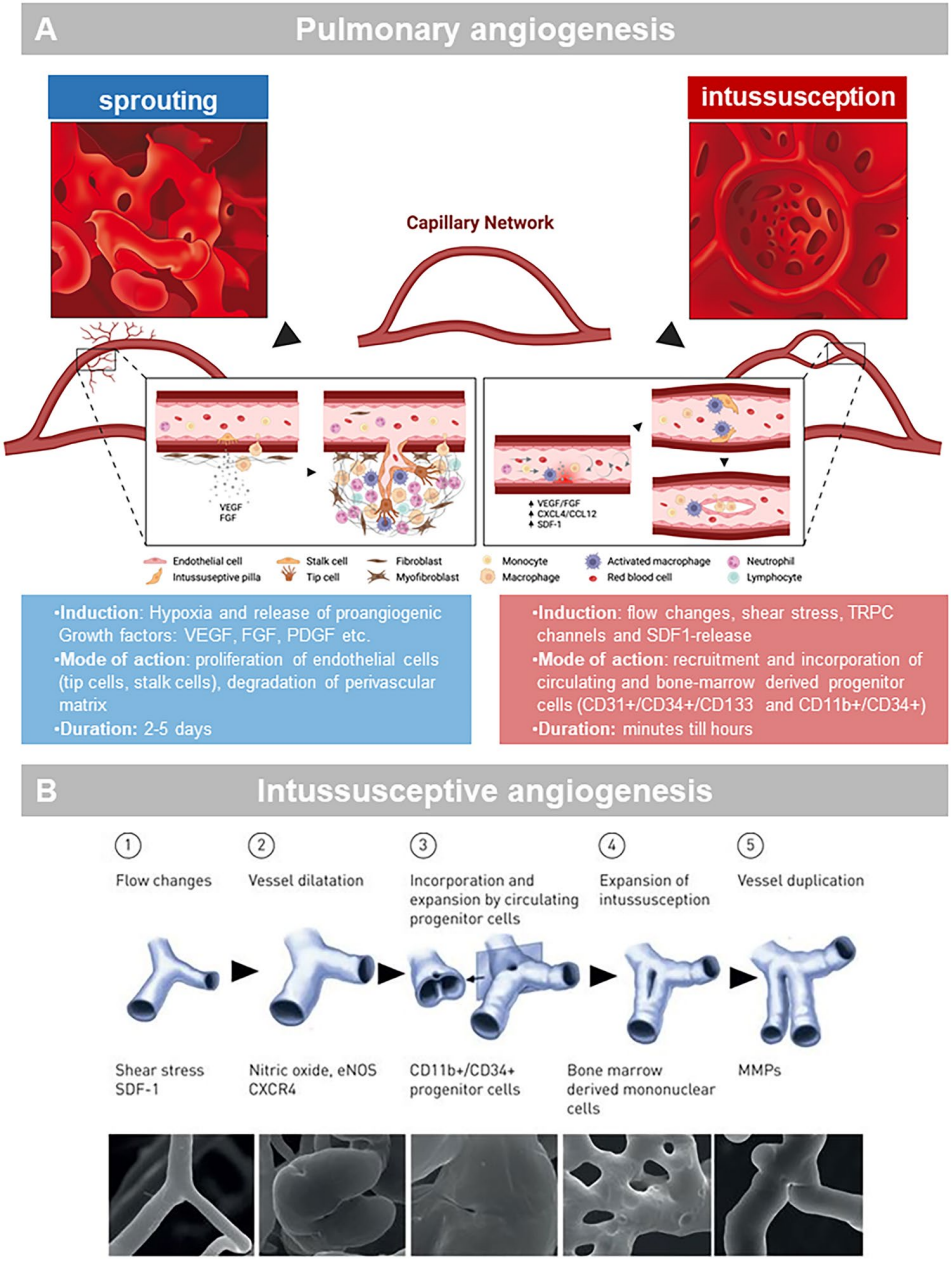
**A** Schematic of pulmonary angiogenesis by sprouting and intussusceptive angiogenesis. **B** Illustrations and microvascular corrosion casting depict the morphogenetic processes during the intussusceptive expansion

The formation and the remodeling of new blood vessels from existing vessels—a process known as angiogenesis (Fig. 1)—occurs in normal lung development as well as in pathological conditions involving inflammatory diseases, pulmonary fibrosis or lung carcinoma. This blood vessel expansion can come either by sprouting or by non-sprouting, intussusceptive angiogenesis [4, 25, 26]. Whereas sprouting angiogenesis tends to follow the gradient of released proangiogenic growth factors (e.g. VEGF, FGF or PDGF), the latter has been described as a well-characterized morphogenetic process which can be observed during growth and remodeling of pre-existing networks by spanning the vascular lumen by a intussusceptive pillar with a diameter of 1–5 µm. Similarly, endothelial colony-forming cells (ECFC) and monocytes have been described to integrate into the vessel wall [25, 27]. These circulating progenitors primarily express endothelial antigens such as CD34, CD11b, Tie2, CD133, and VEGFR-2 and show phenotypic similarities to mature endothelial cells [27]). Although the number of circulating endothelial progenitor cells in a healthy organism is extremely low at 0.002% of all mononuclear cells [28], this cell fraction can increase massively, especially during ischemia and tumor growth. There is evidence that changes in shear stress and micromechanical forces on the endothelial cells are involved in triggering the recruitment of blood-borne progenitor cells by the release of attracting factors (e.g. SDF1, CXCR4, CCL12) [29, 30]. In contrast to sprouting angiogenesis, the formation of new vessel segments by intussusception occurs without active cell proliferation, increased vascular permeability, or cell invasion [31]. Thus, the process of intussusceptive angiogenesis (Fig. 1) represents a highly dynamic intravascular morphological process that can remarkably alter and adapt the structure of the microcirculation within a very short time to maintain the metabolic demands as observed in development [32], regeneration [33], inflammatory processes [34, 35], or tumors [36, 37]. Intussusceptive expansion of the vasculature occurs predominantly in the venous limb or in the capillary bed. However, it can also be observed to a lesser extent in arterioles and smaller arteries [25]. The occurrence of sprouting angiogenesis is accompanied by the differentiation of tip cells and stalk cells [38, 39] in endothelial cells with a high expression of VEGFR2 in tip cells and upregulated levels of DLL4 in stalk cells [40]. After the sprouting initiation, endothelial cell proliferation and a directional migration by the degradation of the surrounding extracellular matrix have been shown to fulfill the stabilization of the newly formed vessel sprouts [41].

## Impact of endothelial precursors for pulmonary angiogenesis

In recent decades, interest in the role of endothelial progenitor cells in angiogenesis has grown rapidly due to their enormous therapeutic potential in cardiovascular medicine and as biomarkers of vascular senescence [42–45]. Since the initial description of "endothelial progenitor cells (EPCs)" by Asahara and coworkers in 1997 [46], who isolated CD34 + /VEGFR2 + endothelial cells from human peripheral blood that formed sprouting angiogenic cell clusters, the ambiguous origin of endothelial progenitor cells remains to be elucidated. Several years later, Yoder and coworkers were able to identify endothelial colony-forming cells (ECFCs), widely accepted as "true EPCs", which represent a vascular resident endothelial cell type with high intrinsic proangiogenic potency [47].

Basically, there is a fundamental distinction between endothelial colony-forming cells (ECFCs) and myeloid angiogenic cells (MACs), both cell populations possessing proangiogenic activities compared to circulating endothelial cells (CECs), which are detached mature endothelial cells with limited proangiogenic capacity [44]. The derivation of endothelial colony-forming cells (ECFCs) from non-hematopoietic sources [47] is thought to be highly proliferative cells that form tube-like structures or sprouts, whereas myeloid angiogenic cells (MACs) are characterized as bone marrow-derived cells that are non-proliferative, non-sprouting cells that support angiogenesis by intussusception or by paracrine effects [48]. Several factors have been shown to stimulate the functional activity of ECFCs: e.g. SDF1 [49], VEGF-A [50], TGFβ1 [51], thrombin receptor PAR1 [52], nestin [53] or osteoprotegerin [54]. In principle, ECFCs can be used therapeutically to improve vascularization in chronic lung diseases. On the other hand, they also offer the possibility to monitor the course and progression of many lung diseases such as COPD [55] (or pulmonary fibrosis [56, 57]. While there is no clinical evidence of a potential therapeutic benefit of treating chronic respiratory diseases with ECFCs, the diagnostic value of ECFCs as a biomarker for chronic respiratory diseases has been elucidated [58, 59].

Chronic obstructive pulmonary disease (COPD) and idiopathic pulmonary fibrosis (IPF) are two severe chronic lung diseases characterized by distinct clinical and pathological features. In COPD, pulmonary emphysema is characterized by loss of alveolar structure and endothelial cells. Paschalaki and coworkers demonstrated accelerated senescence in smokers and patients with COPD compared to non-smokers [60]. Furthermore, it is now well recognized that patients with COPD have heterogeneous skeletal muscle responses to exercise due to defective pericyte coverage and dysfunctional capillaries [61]. In pulmonary fibrosis, Smadja and colleagues investigated the impact of ECFCs as a fluid biomarker in patients with idiopathic pulmonary fibrosis (IPF). They observed increased senescence and a higher frequency of apoptosis of ECFCs isolated from IPF patients compared to age-matched healthy controls [62], with the number of ECFCs being significantly higher in patients with impaired diffusing capacity (DLCO). In addition, they provide evidence that microparticles released from ECFCs isolated from IPF patients could stimulate fibroblast migration and activation through increased levels of plasminogen and interleukin 8 ([57, 62]. Compared to stable disease, ECFC proliferation was increased in patients with exacerbations of IPF [56] or reduced lung function in systemic sclerosis-interstitial lung disease (SSc-ILD) [63]. Clinical data suggest a crosstalk between ECFC proliferation and fibrocytes through activation of the SDF-1/CXCR4 pathway [49]. Furthermore, ECFC proliferation is associated with angioproliferative processes in pulmonary arterial hypertension (PAH), which is characterized by progressive pulmonary vascular remodeling and precapillary pulmonary hypertension [64]. There is compelling evidence that ECFC from pulmonary arterial hypertension patients with BMPR2 mutations exhibit a vasculoproliferative phenotype without the formation of stable vascular networks, highlighting the importance of ECFC in the pathogenesis of PAH [64, 65]. In pediatric patients with PAH treated with treprostinil, a prostacyclin vasodilator. ECFC proliferation was stimulated by treprostinil-mediated release of VEGF-A by mesenchymal stem cells [66]. It might be expected moreover that ECFC will be further be studied as a surrogate marker for pulmonary angiogenesis in chronic lung diseases. Thereby, larger patient cohorts should elucidate the mechanisms of disease progression in COPD, lung fibrosis or pulmonary hypertension.

## Bacterial and viral respiratory diseases

Infectious airway diseases, especially lower respiratory tract infections are still a prevalent disease and a leading cause of death, particularly in children and the elderly with a high incidence of ~ 6.000 per 100.000 and a mortality rate of 34.3 per 100.000 placing them in the top five of causes of death [67, 68]. Clinical symptoms range from mild bronchitis (e.g. common cold), pneumonia with fever and respiratory distress to severe acute respiratory distress syndrome (ARDS), sepsis and multi-organ failure. Causative agents are primarily pneumotropic bacteria (e.g. staphylococcus pneumonia), viruses (e.g.influenza viruses, respiratory syncytial viruses, adenoviruses and coronaviruses) and to a lesser degree fungi (e.g. pneumocystis jiroveci, aspergillus species). As most infectious pathogens are airborne, the respiratory epithelium often is the primary site of host-reaction and inflammation to the infectious agent and has been studied extensively [69]. More recently, notably due to recent viral pandemics of H5N1 influenza A virus, SARS-CoV-1, MERS and especially SARS-CoV-2, the vascular system and endothelial cells as one of the most abundant cell types in the lung, as well as extrapulmonary effects of pulmonary infectious diseases became a focus of interest.

Several bacteria responsible for manifest pneumonia can modulate the inflammatory state of endothelial cells, disrupt the endothelial barrier and cause direct endothelial cell damage affecting leukocyte adhesion, cytokine-secretion and thus manipulating leukocyte and especially neutrophil migration (e.g. pneumolysin of Streptococcus pneumonia, shiga toxin 2 of e.coli or ExoU of Pseudomonas aeruginosa). Further, the bacterial toxins can cause direct damage to vascular smooth muscle cells (e.g. lethal toxin of bacillus antracis) and cause indirect vascular leakage through neutrophil-activation (e.g. phenol-soluble modulin a4 of S: aureus) [70]. These mechanisms lead to a disruption of lung endothelial homoeostatic mechanisms propagating lung parenchymal inflammation and thus increasing disease severity [17, 71]. Despite these vascular alterations, bacterial infections of the lung are mostly characterized by direct parenchymal and especially epithelial injury with edema, a classic pro-inflammatory cytokine milieu and pathogen driven adaptive immune response. They usually resolve in a restitution ad integrum with rarely chronic residues, e.g. in form of interstitial lung disease often in form of organizing pneumonia [72], often not easily differentiated from ventilator associated lung injury and interstitial fibrosis after ARDS, independent of the causative agent [73]. Circulating endothelial progenitor cells, a sign of pulmonary repair (see below), are increased in patients with pneumonia, but interestingly tend to be lower in cases of persistent fibrotic changes hinting at impaired repair mechanisms [74] also discussed for other fibrotic interstitial lung diseases [75] with neovascularization and repopulation with endothelial cells being important components of repair and wound healing (see below). However, recruitment of local and circulating endothelial progenitor cells seems to be a general pattern in ARDS not limited to bacterial infection and with a beneficial clinical course often displaying higher number of circulating endothelial progenitor cells than non-survivors. These findings are corroborated by several animal studies of acute lung injury [76]. In a parabiosis model, significant recruitment and incorporation of peripherally circulating CD34 + endothelial progenitor cells was demonstrated during compensatory lung growth [77].). A possible cause of this strong cell activation and migration could be the dramatic vascular expansion by intussusceptive angiogenesis [25, 77]. In rodents and humans, it has been shown that there is a residual population of endothelial progenitor cells in the lung that can differentiate de novo into blood or lymphatic endothelial cells [78]. These residual endothelial progenitor cells are thought to play a pivotal role in the healing process of acute lung failure or ARDS (Rafat et al., 2013). A similar increase in circulating progenitor cells has also been described in patients recovering from bacterial pneumonia [74].

While the effect on the vascular system and endothelial cells in bacterial infections is mostly of indirect nature and rather an effect of the induced pro-inflammatory response, several viral agents – especially SARS-CoV-2, tend to induce a more vasculocentric pathomechanism. However, with the highly pathogenic avian influenza strain H5N1 being an exception, influenza virus have not shown direct infection of endothelial cells [79, 80], the IL-6 dominated inflammatory milieu leads not only to endothelial permeability and barrier dysfunction with concomitant pulmonary edema and exudation, but, through activation of tissue factor, leads to a pro-thrombotic milieu and, dependent on various factors either activation of the innate immune system and decreased viral replication through protease-activated receptor 2 or increased inflammation and virus replication via protease-activated receptor 1 [81]. Consistently, viral-associated thrombotic microangiopathies have been described in numerous inflammatory cardiorespiratory diseases including influenza [35, 82]. Interestingly, influenza infection increases Pattern Recognition Receptors with resultant endothelial leak and apoptosis following exposure to Staphylococcus aureus-derived PAMPs in vitro explaining the often observed clinical worsening and threat of bacterial superinfection in influenza pneumonia [83].

**Fig. 2:**
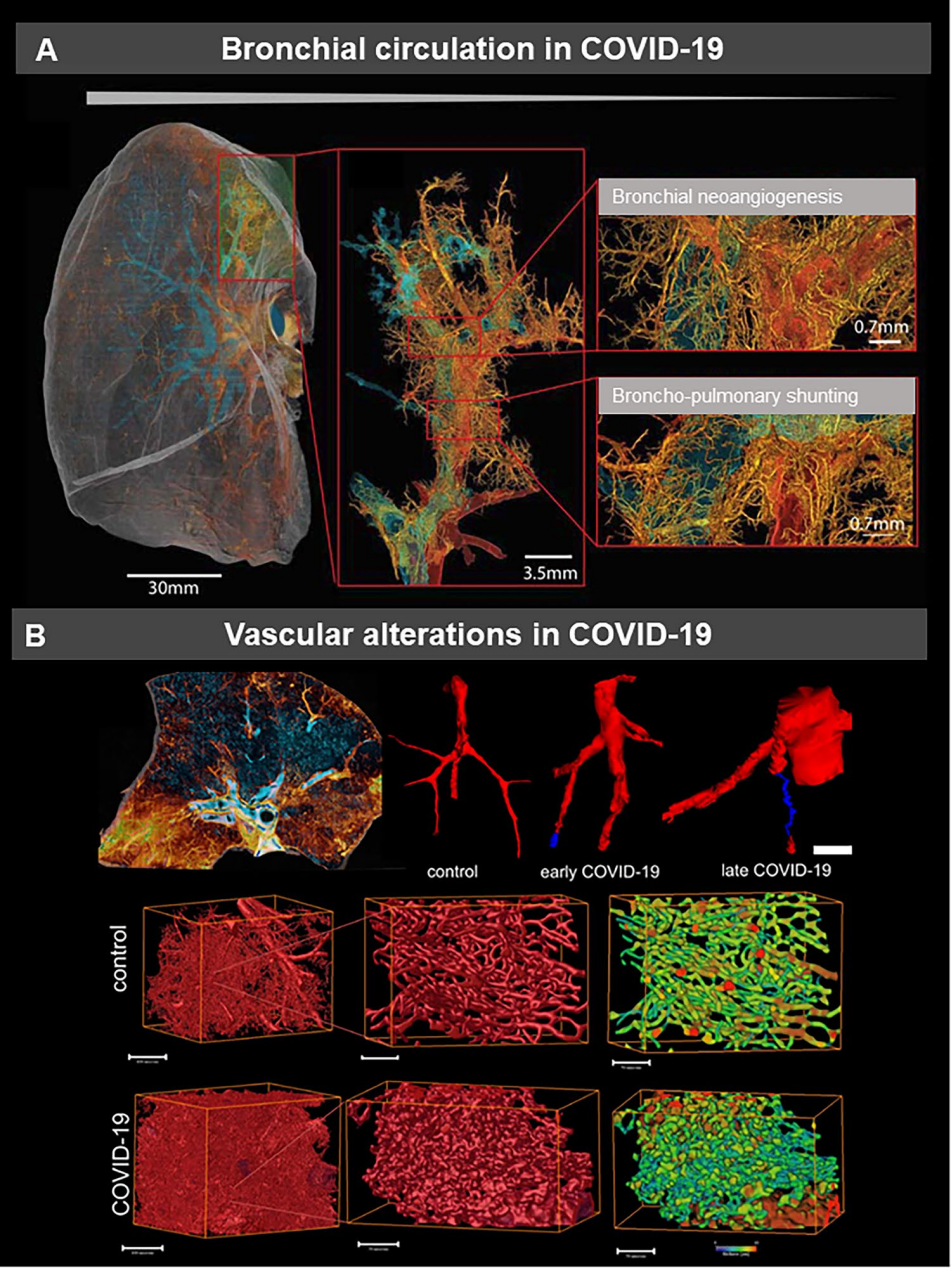
Multi-resolution imaging of a COVID-19 autopsy lung, using hierarchical phase contrast tomography (HiP-CT) illustrates the vascular remodeling of the bronchial circulation COVID-19 patients. Microvascular changes in COVID-19 lungs: **a** Volume rendering of a representative hierarchical phase-contrast tomography (HiP-CT) slice shows the spatial heterogeneities of microischemia and fibrotic remodelling of the airways. Micro-CT-based 3D reconstruction of subsegmental pulmonary arteries (red) and airways (blue) showed (sub-)total arterial occlusion in COVID-19 lungs of early and late hospitalized patients compared to uninfected controls **c** Three-dimensional evaluation of microvascular corrosion casts by synchrotron radiation tomography microscopy illustrating the altered and increased alveolar vascularity in COVID-19 lungs

This shift to a more endothelial and thus vascular driven infection becomes even more apparent in the instance of COVID-19 (Fig. 2), a genuine multi-organ vascular disease rather than a respiratory infection alone. Compared to influenza and other causes of ARDS, COVID-19 lungs at autopsy of patients succumbing to their disease are significantly lighter, hinting at less edema and frequently show parenchymal and intraluminal blood clots, a sign of hypercoagulability [84] or immunothrombosis by the formation of dysregulated neutrophil extracellular traps (NETs) [85]. These findings explain the often observed, so called, “silent hypoxia” [86] in COVID-19 compared to influenza with the latter commonly displaying shortness of breath and elevated work of breathing [87]. Mechanistically, COVID-19, in contrast to influenza and other conventional viral inflammatory reactions, is characterized by an angiocentric and especially CD4 + T-helper cell dominated inflammation with prominent endothelialitis, in part due to direct infection of endothelial cells by SARS-CoV-2 [88]. These lead to swelling and disruption of the endothelial cell barriers, an anomalous microvascular architecture (Fig. 2), and an endothelial dysfunction [89, 90]. The result is a ~ nine fold increase in capillary microthrombi and a ~ 30-fold increase in intraluminal pillars and split blood vessels on vascular corrosion casting, a hallmark of a special form of angiogenesis termed intussusception [84].

The consequence is not only ineffective gas exchange, but also tissue ischemia. Localized tissue ischemia within the lung is normally compensated by the bronchial circulation a network branching off the descending aorta to perfuse the proximal airways as well as the fibrous septa of a distinct, rather vascular, than airway defined, anatomical structure called the secondary lobule [91]. The bronchial circulation is sought to prevent local tissue ischemia. Knowledge gained from observations in lung transplantation and airway reconstructive surgery where the bronchial circulation is disrupted indicates that if concurrent pulmonary artery embolism occurs, this leads to hemorrhagic pulmonary infarctions [92]. Recently, through high resolution Synchrotron radiation based hierarchial phase-contrast tomography (HiP-CT) we could demonstrate a mosaic-like consolidation of individual secondary pulmonary lobules based on microvascular occlusion and secondary lobular microischemia [93], potentially explaining the described increased alveolar dead space [16]. Furthermore, in severe COVID-19, we could observe an increase of intrapulmonary left–right-shunts, the so called “Sperrarterien”, leading to expansion and dilatation of the bronchial plexus [10].

Thus, the long-term, post-acute sequelae of a COVID-19, defined as post-acute COVID-19 syndrome (PACS) beyond the 4 weeks of acute infection, are characterized by a persistent systemic thromboinflammation with complement dysregulation [94] and a pulmonary microischemia-induced fibrotic remodelling to COVID-19. Although similarities between the histologic presentation and genetic background of PACS-associated pulmonary fibrosis and UIP have been reported [95], own data [96] show that gene expression, plasma protein expression, and disease course in COVID-19 are distinct from the common forms of interstitial lung diseases, including, e.g. key genes related to matricellular markers (GDF15, CD163), angiogenesis (CXCR4, CXCL12), and fibrosis (MMP1, COL3A1) which may underline the unique vascular etiology of COVID-19 [97]. There is recent clinical evidence of increased serum levels of VEGF-A, von Willebrand factor (vWF) [98], calprotectin [99], fibrin monomers [100] among others, in PACS patients as a relevant predictive biomarker for impaired lung function and radiologically assessed lung consolidation [101]. The markedly elevated levels of angiopoetin-1 (ANG1) in PACS patients may underline a long-term, wound-repairing angiogenic response due to the SARS-CoV2-associated endothelial injury [102], whereas increased angiopoietin-2 (ANG2) levels were associated to the fatal trajectory of COVID-19 reflecting the systemic microangiopathy by vascular disruption and remodeling in hospitalized COVID-19 patients ([96, 103].

Taken together, severe COVID-19 is characterized by an angiocentric system and organ-spanning inflammation, prompting pulmonary and vascular compensation and repair mechanisms (intussusceptive angiogenesis, expansion of bronchial circulation and activation of left-to-right shunting) to such a degree and expanded time-span that it ultimately results in a downward-spiral increasing local (pulmonary) tissue and systemic hypoxia thus worsening the cause of disease and further bringing the lung at risk of consecutive irreversible fibrotic remodeling [96].

## Chronic obstructive lungs diseases (COPD)

**Fig. 3:**
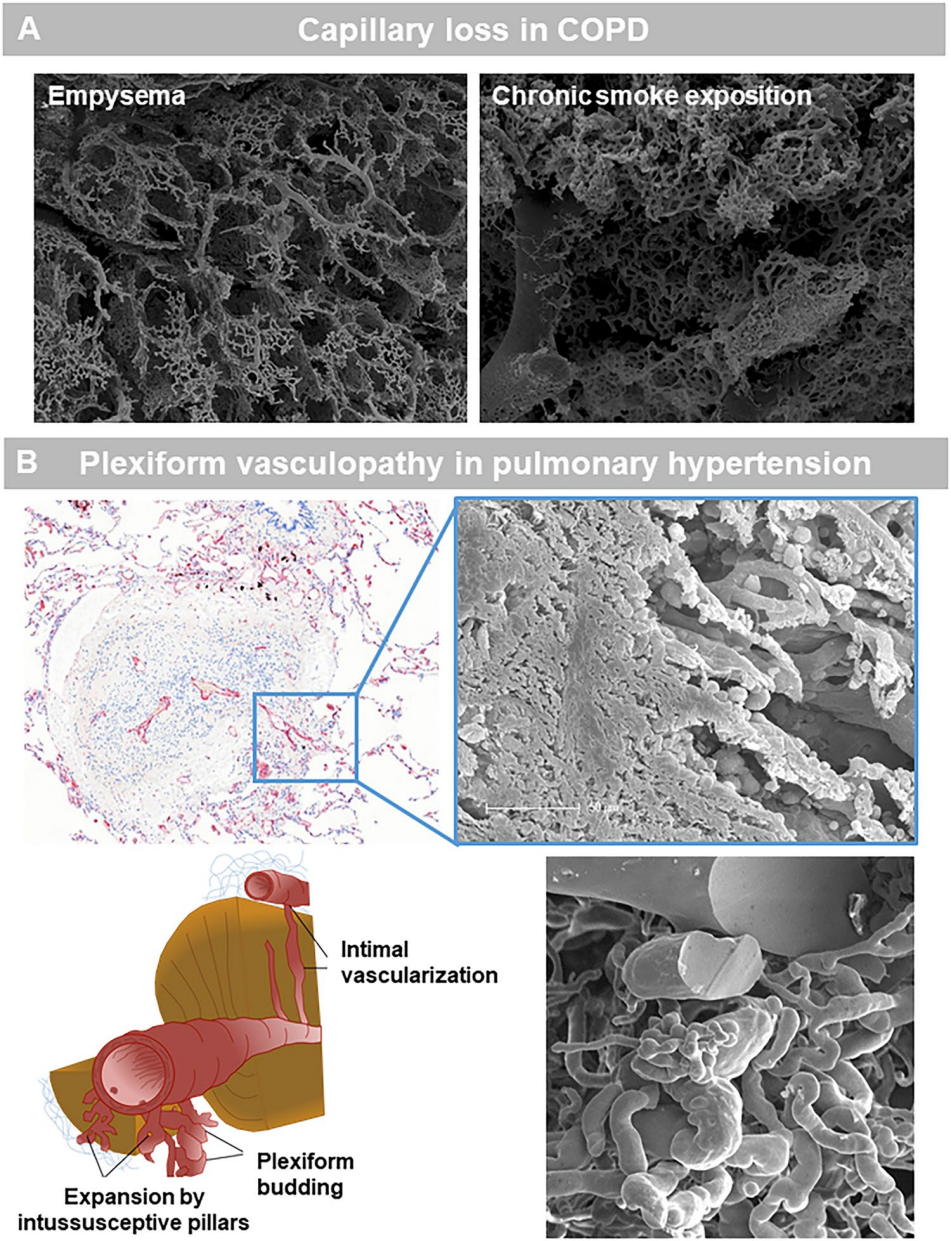
Scanning electron micrographs of microvascular corrosion casting of an elastase-induced emphysema model in mice (left) and chronic smoke exposition (right) in a murine smoke chamber model depict the capillary loss in emphysema and a peribronchial vascular remodeling. In pulmonary hypertension, anti-CD31 positive vessels depicts a microvascular outgrowth which is a morphological characteristic of plexiform lesions (SEM in blue box). A schematic illustration shows the expansion of vascular plexus in pulmonary hypertension. Scanning electron micrographs of microvascular corrosion casting of a PH lungs shows a plexiform budding with a loss of vascular hierarchy

COPD is the most common chronic respiratory disease and the third leading cause of mortality according to the WHO. It is characterized by destruction of the lung parenchyma, small airway obliteration and loss. Mild to moderate pulmonary hypertension is a common morbidity in COPD patients, although a small fraction of the patients develop severe pulmonary hypertension. This led to the definition of a pulmonary vascular phenotype of COPD [104], which is relatively rare and is characterized with severe pulmonary hypertension (PH) and low diffusing capacity of the lung for carbon monoxide, but only mild-to-moderate airway obstruction. Typically, these patients show medial hypertrophy and intimal thickening, with the degree of remodeling depending on disease severity. Endothelial dysfunction/loss is believed to be an early event in COPD pathogenesis (Fig. 3). Indeed, in mild COPD intimal thickening, especially in smaller arteries can already be observed [105]. A reduction in capillary length and density was also found in COPD patients relative to controls [106]. Therefore, (micro)vascular dysfunction and remodeling are typically associated with emphysema, and the decrease of the capillary bed surface area is thought to be linked to dysregulated angiogenesis and endothelial apoptosis [107]. In-vivo imaging confirms that decreased lung perfusion, increased vessel tapering, and altered large vessel and cardiac structure are evident even at early stages of COPD [108, 109]. Transcriptomic analysis of COPD lung tissue demonstrates a direct relationship between the loss of expression of several endothelial markers (e.g.VEGFR2 and VEGFA) and lung function reduction and increased radiographic evidence of emphysema [110]. A recent single cell sequencing study demonstrated no differences in proportions of endothelial cells between end-stage COPD patients and controls, although an increased gene expression signature was found for cytokine signaling and response to cytokines and stress in the endothelium of COPD patients, where the capillary endothelial cell was believed to be a major contributor to alveolar inflammation [111]. Proof of concept data of the importance of the vascularity in COPD is provided by intravenous delivery of healthy lung endothelial cells in a elastase injury model of emphysema which showed complete rescue of alveolar destruction. Interestingly, emphysema is characterized by a loss of alveolar microvessels due to the loss of vascular homeostasis. The capillary length and length density were significantly decreased to about of 68% control values in a sterological autopsy study by Wiebe and coworkers [106]. There are also several reports that link the vascularity directly to the typical small airway remodeling as seen in COPD via VEGF [112]. The first histologic description of vascular pathology in COPD by Liebow [113] dates 60 years back. Since then more light could be shed on hypoxia-induced vessel remodeling [114], the presence of endothelial cell apoptosis [115] and endothelial cell dysfunction [105, 116] as well, more recently, the existence of hypoxia activated macrophages inducing pulmonary arteriolar smooth-muscle-cell growth via FIZZ1 [117, 118]. Despite these accomplishments, our understanding of how immune cells and their cytokines shape pulmonary vascular remodeling is still in its infancy [119, 120].

### Pulmonary hypertension

Pulmonary hypertension (PH) includes a group of fatal clinical entities such as pulmonary arterial hypertension (PAH) and chronic thromboembolic PH (CTEPH) characterized by dysfunctional angiogenesis leading to pulmonary vascular obliteration in distal-muscular type arteries and consequently to hypertrophy and remodeling of the right ventricle [121]. The histopathology of iPAH includes marked medial, intimal, and adventitial arterial thickening. These remodeled arteries end up as plexiform lesions, consisting of vascular channels lined by a continuously proliferating endothelium and backed by a uniform myogenic interstitium which may arise from anastomoses between the bronchial and pulmonary circulation [122]. Recently, Westoo and colleagues proposed to subdivide the plexiform lesions in 4 distinct classes, either localized within or derived from monopodial branches or localized between pulmonary arteries and larger airways or as spherical structures at unexpected ends of distal pulmonary arteries; or as occluded pulmonary arteries with recanalization [123]. Although iPAH is primarily a disease of the pulmonary circulation, the bronchial circulation could also be involved as individuals with PAH often show evidence of bronchial artery hypertrophy and prominent vasa vasorum in the pulmonary artery [124], while recently also further histological evidence was provided for the role of the bronchial circulation in PAH remodeling and plexiform lesion formation [14]. Pulmonary veno-occlusive disease (PVOD) is also categorized as a type I PH disease, although in a different subclass (1'). This is because PVOD and PAH share a similar clinical presentation, with features of severe precapillary PH. It is however important to differentiate these two conditions as PVOD carries a worse prognosis and PVOD is preferentially involving the pulmonary venous system, although also the capillaries and arteries are affected [125]. In PVOD, intimal remodeling of veins and venules may range from loose fibrous tissue with variable cellularity to dense, paucicellular, sclerotic lesions. Of note complex plexiform lesions are absent in PVOD lungs. Whether pulmonary capillary haemangiomatosis (PCH) is a distinct entity from PVOD remains controversial. There is typical abnormal capillary proliferation within the alveolar interstitium, but there are many overlapping features with PVOD including overlapping mechanisms [126, 127]. Our own recent findings in PVOD suggest that venous occlusions in PVOD increase shear stress and micromechanical forces leading to an expansion of intussusceptive angiogenesis [127]. Chronic thrombo-embolic pulmonary hypertension (CTEPH) is the most prominent disease in type IV PH. In CTEPH, blood clots extend within the artery, cling to the artery wall and resembles scar tissue, usually starting in large or middle-sized pulmonary arteries. This increases pulmonary pressure, reduces blood flow into the lungs and can trigger narrowing of other blood vessels throughout the lung. Small-vessel disease can concomitantly occur in obstructed areas, possibly triggered by unresolved thrombotic material, and downstream from occlusions, possibly because of excessive collateral blood supply from high-pressure bronchial and systemic arteries [128]. Plexiform lesions can also be observed in CTEPH [129] (Fig. 3). Anastomoses between bronchial artery branches and precapillary pulmonary arterioles appear during evolution of the disease. Other acquired vascular connections between bronchial arteries and pulmonary veins may also trigger venous remodeling [130].

## Interstitial lung diseases (ILD)

**Fig. 4:**
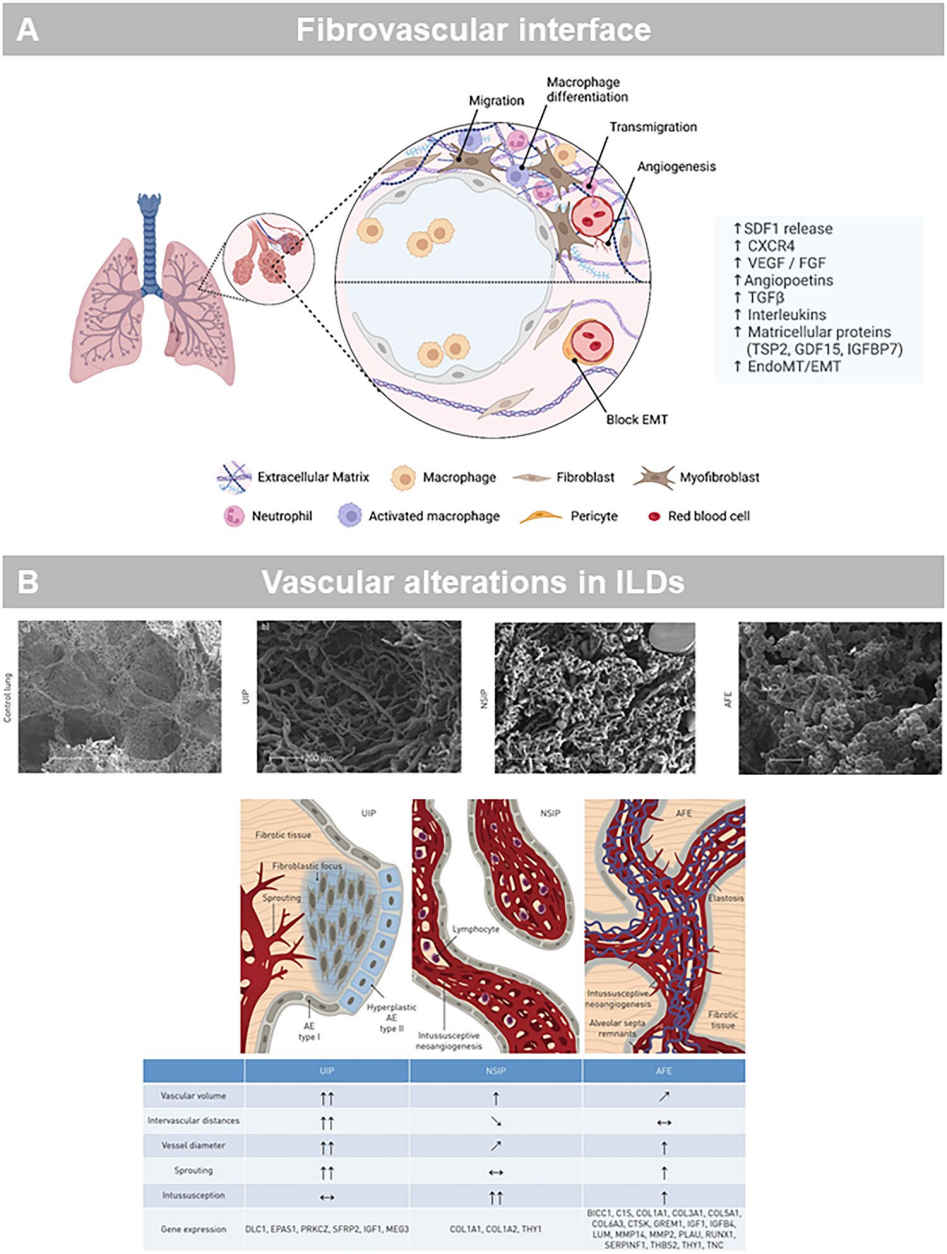
**A** Schematic illustrates the fibrovascular interface in fibrotic lung diseases. **B** Scanning electron micrographs of microvascular corrosion casts illustrate the substantial architectural differences between the different injury patterns. Healthy control lung vasculature is characterized by thin-walled alveolar capillary plexuses aligned along the alveolar duct; UIP lungs demonstrate an aberrant vasculature with blunt, sinusoid-like vessels, without a clear vessel hierarchy, but instead high variability of vessel diameters and small vessel sprouts; NSIP lungs present with dense, tortuous dilated tufts of vessel formations in the alveolar septa and frequent intussusceptive pillars; AFE lungs resemble the appearance of the NSIP lungs with pronounced vascular alterations in the alveolar septa. Schematic illustration of morphomolecular motifs in histopathological subtypes of interstitial lung injury models

Interstitial lung diseases encompasses nowadays diverse and complex group of over 300 non‐neoplastic pulmonary diseases with distinct morphological and molecular motifs [131]. Currently, several interstitial injury patterns are indicative, but not pathognomonic for any given ILD entity among these variable morphologies [131]. The spatial and temporal heterogeneity of various pulmonary remodeling results in an overlap of injury patterns among the most common: usual interstitial pneumonia (UIP), non‐specific interstitial pneumonia (NSIP), organizing pneumonia (OP) and alveolar fibroelastosis (AFE) [75] (Fig. 4). The characteristic feature of the UIP pattern is spatially and temporally heterogeneous interstitial fibrosis driven by aggregates of myofibroblasts, so-called fibroblast foci, with secondary honeycomb alterations, smooth muscle metaplasia and proliferation. In those UIP-lungs, we observed a higher density of the upstream vascularity and perifocally blind-ending vessels with a loss of hierarchy. Another relatively common injury pattern that has only recently been identified and defined is AFE, also known as pleuroparenchymal fibroelastosis (PPFE) in certain clinical situations. Histologically, AFE shows intra-alveolar collagenous obliteration with discrete accompanying inflammation and dominant elastosis in the adjacent former alveolar walls [75]. Currently, it has been hypothesized whether angiogenesis could be considered as an initial trigger in the the pathogenesis and the progression of pulmonary fibrosis [75, 132, 133].

In particular, the alveolar–capillary permeability is abnormally increased in patients with pulmonary fibrosis by the release of pro-inflammatory and pro-angiogenic mediators [134] which is accompanied by microvascular injury, turbulent flow, microthrombi and an alveolar-vascular remodeling [135]. The microvascular injury is mainly specified by an alteration of vascular homeostasis and loss of endothelial markers as platelet endothelial cell adhesion molecule (PECAM)-1 and vascular endothelial cadherin (VE-cadherin) [136] leading to higher transmural shear stress and a flow-induced release of nitric oxide by the stimulation of endothelial nitric oxide synthase (eNOS) [137] or higher expression of stromal cell-derived factor-1 (SDF-1) by activating calcium-dependent transient receptor potential (TRP) in endothelial cells [138–140]. Thereby, the stimulation of the haematopoetic-vascular niche results in the angiocrine recruitment of monocytes (CD11b/TIE2^+^) and circulating proangiogenic cells (CD31^+^/CD34^+^/CD133^+^) [27, 30, 141] or the activation of perivascular macrophages (VEGFR1^+^) or perivascular fibroblasts (Jag1^+^) [142]. Thus, perivascular macrophages, monocytes and circulating progenitor cells are key regulators of angiogenesis in the fibrovascular interface (Fig. 4) in health and disease and are involved in different stages of fibrovascular remodeling, leading to different vascular architectures in health and in ILD [75, 143, 144] (Table 1).

**Table 1:** Histologic features of lung diseases relevant to intussusceptive angiogenesis

	Fatal hypoxia	Lymphocytes	Endothelialitis	Microthrombi	Intussusceptive Angiogenesis
Influenza A^a^	Yes	Yes	–	–	↔
Endstage NSIP^a^	Yes	Yes	–	–	↑↑
AFE^a^	Yes	Yes	–	–	↑↑
UIP^a^	Yes	–	–	–	↔
CTEPH^c^	Yes	–	Yes	Yes	↑
PCH^d^	Yes	–	Yes	Yes	↑
PVOD^d^	Yes	–	Yes	Yes	↑
COVID-19^a^	Yes	Yes	Yes	Yes	↑↑↑

^a^Ackermann M, Verleden SE, Kuehnel M, Haverich A, Welte T, Laenger F, Vanstapel A, Werlein C, Stark H, Tzankov A, Li WW, Li VW, Mentzer SJ, Jonigk D. Pulmonary Vascular Endothelialitis, Thrombosis, and Angiogenesis in Covid-19. N Engl J Med. 2020 Jul 9;383(2):120–128

^b^Ackermann M, Stark H, Neubert L, et al. Morphomolecular motifs of pulmonary neoangiogenesis in interstitial lung diseases. Eur Respir J 2020;55

^c^Ackermann M, Gaumann A, Mentzer SJ, et al. Plexiform vasculopathy in chronic thromboembolic pulmonary hypertension. Am J Respir Crit Care Med 2017;196:48–51

^d^Neubert L, Borchert P, Shin HO, Linz F, Wagner WL, Warnecke G, Laenger F, Haverich A, Stark H, Hoeper MM, Kuehnel M, Ackermann M, Jonigk D. Comprehensive three-dimensional morphology of neoangiogenesis in pulmonary veno-occlusive disease and pulmonary capillary hemangiomatosis. J Pathol Clin Res. 2019 Apr;5(2):108–114. doi: 10.1002/cjp2.125. Epub 2019 Feb 27

In human ILD samples, alveolar-vascular remodeling is mainly characterized by the occurrence of intussusceptive and sprouting angiogenesis as morphological hallmarks of the respective interstitial injury [75]. NSIP lungs were dominated by higher frequencies of pillar formations in comparison to UIP and AFE lungs, whereas sprout formation was 10 times more frequently in UIP lungs compared to NSIP and AFE lungs. Recent observations [21, 145] could identify a dramatic increase in a COL15A1^+^-endothelial cell population and a replacement of alveolar capillaries with COL15A1^+^ blood vessels in human NSIP lungs in which a high frequency of intussusceptive angiogenesis was predominantly observed in the expanded interlobular septa and in the subpleural spaces [75]. COL15A1^+^ blood vessels were localized in systemic-venous endothelial cells in the bronchial circulation or in the visceral pleura [21]. It appears remarkable that the cellular expansion of ectopic peribronchial endothelial cells is predominantly involved in the fibrovascular remodeling in IPF patients [146]. The pivotal role of bronchial circulation in lung fibrosis has been already hypothesized by Turner Warwick in 1963 who observed an increased perfusion by intrapulmonary bronchopulmonary anastomoses in patients with fibrosing interstitial lung diseases [147].Given the significance of angiogenesis and vascular remodeling in repair processes, it could be controversially discussed whether angiogenesis [148, 149] acts as double-edged sword, on the one hand in restoring the microcirculation and the passage of lymphocytic cells and progenitor cells, on the other as triggering factor of disease progress by aberrant, dysfunctional bundles of blood vessels which could be transformed to myofibroblasts by endothelial-mesenchymal transition (EndoMT) [150]. This crucial question can only be addressed by spatial dependence and temporal heterogeneities observed in the morphology of interstitial lung diseases with a heterogenous distribution or UIP/NSIP overlap [131]. So far, nintedanib is the only clinically approved tyrosine kinase inhibitor for idiopathic pulmonary fibrosis (IPF), progressive pulmonary fibrosis or systemic sclerosis–associated interstitial lung disease (SSc-ILD) which significantly reduced the decline in lung function and the disease progression [151–153]. In addition to the anti-inflammatory and anti-fibrotic effects, nintedanib inhibits the proliferation of numerous pulmonary vascular cell types [152] and leads to restoration and ‘normalization’ of the altered vascular architecture [154], a remarkable structural finding observed in anti-angiogenic therapy in tumors [36, 155]. Taken together, the close interaction of pulmonary neoangiogenesis with fibrogenesis suggests that the fibrovascular interface is a decisive factor for the progression and aggravation of pulmonary fibrosis.

### Lung cancer and angiogenesis

**Fig. 5:**
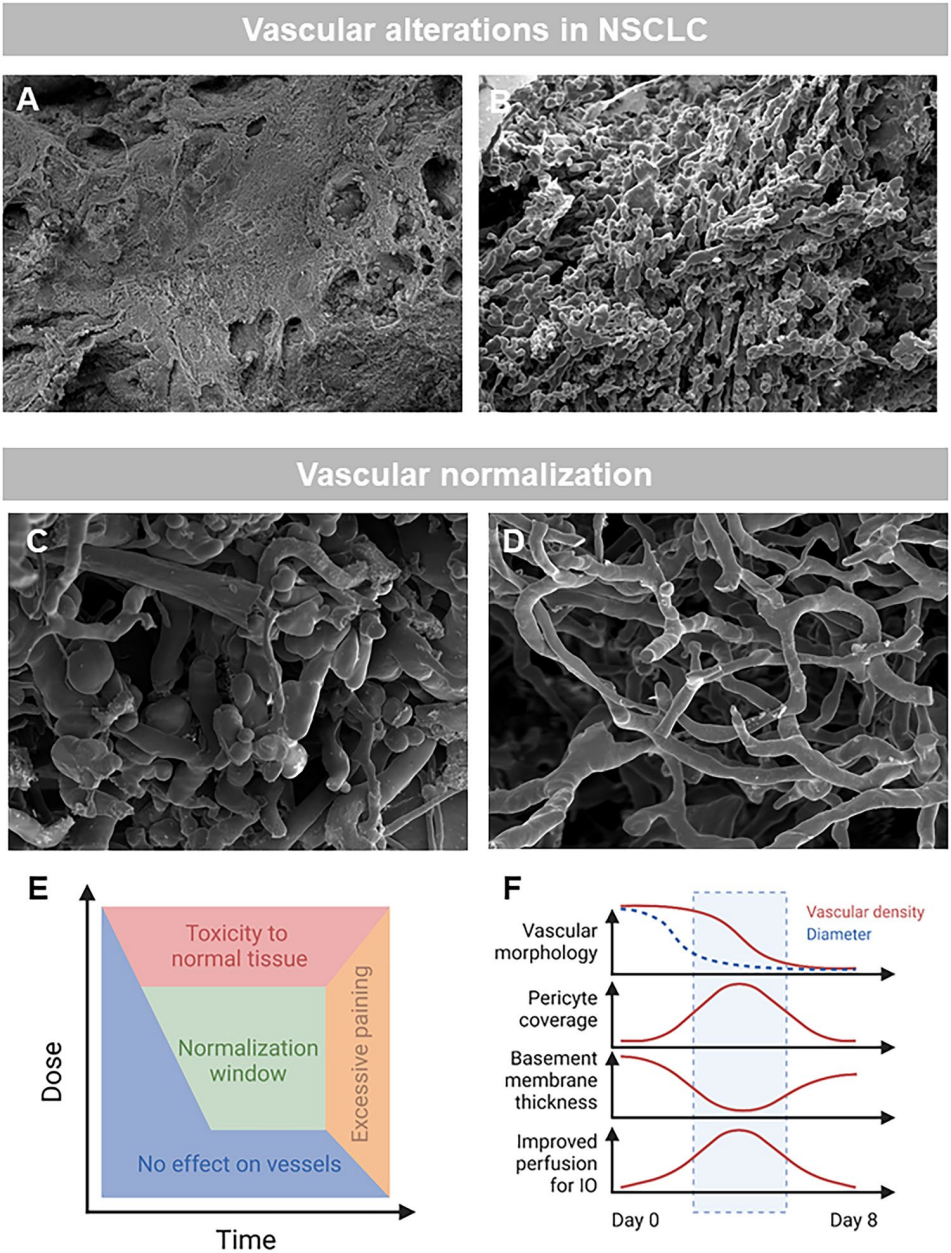
**A** Scanning electron micrograph shows the cross-section of a human pulmonary adenocarcinoma. **B** Microvascular corrosion casting depicts the vascular architectural alterations in the before-mentioned pulmonary adenocarcinoma characterized by abnormal, tortuous blood vessels with a missing hierarchy. **C** Scanning electron micrograph of the microvascular corrosion casting in A549-NSCLC-xenografts shows an abnormal tumor vascularity with blind-ending vessels and elongations. **D** In bevacizumab-treated A549-NSCLC- xenografts, the tumor vascularity is restored to a “normalized” architecture. **E** The transient normalization window results in a higher vulnerability of cancer cells to cytotoxic agents and an improved perfusion of immune checkpoint inhibitors. **F** Effects of anti-angiogenic treatments on vascular morphology and angio-immunlogic switch

The strict relationship between tumor progression and vascularization was postulated as early as 1971 by Judah Folkman in his hypothesis of the "angiogenic switch" in the New England Journal of Medicine [156]: A solid tumor with a volume greater than of more than 1–2 mm^3^ is absolutely dependent on the formation of new blood vessels. The tumor tissue switches to a "proangiogenic phenotype" and releases proangiogenic growth factors, which promote further vascularisation of the advancing tumor [157]. All organs and tissues have a very specific vascular architecture that reflects the needs of the organ and its metabolism. Ultimately, the design principle applies: "form follows function". In many tumors, however, this organ- and tissue-specific vascular architecture is not preserved. In the newly formed tumor vessels, the clear autochthonous vascular hierarchy of arterioles, capillaries and venules is largely abolished [158]. The vascular architecture shows a chaotic arrangement of various blind ending vessels with a predominantly aberrant, tortuous course [36] (Fig. 5 A,B). Sinusoidal tumor vessels often form a “rete mirabilis” in which not only venous drainage but also capillary elongations with vessel breaks are found, which can increase the vessel density many times. However, this abnormal vascular network makes little functional contribution to nutritive blood flow, which explains the low intratumoral oxygen partial. The permeability in these vessels is also increased due to endothelial fenestration. In the newly formed tumor vasculature, this results in gaps in the endothelial cell population of approximately 200 nm–2 µm [159]. These ultrastructural changes lead to a fluid shift into the tumor interstitium, which can increase the interstitial pressure by 20–50 mmHg [160]. The consequence of this increased interstitial pressure in the tumor is that the poor, heterogeneous perfusion of the tumor significantly impedes the absorption of chemotherapeutic agents. It also increases lymphatic drainage in the tumor, which increases the likelihood of lymphogenic metastasis [161]. It should be noted that the characteristic phenomena of tumor vessels may occur to a lesser extent in precancerous lesions even before malignant progression [162]. Nevertheless, studies of pulmonary angiogenesis and its role in lung neoplasia appear to have lagged behind those in other organs perhaps given the complexity of the lung with its two different circulatory beds as discussed previously [163]. Although numerous anti-angiogenic treatments including anti-VEGF antibodies (e.g. bevacizumab), anti-VEGFR-2 antibodies (e.g. ramucirumab) or multi-kinase inhibitors targeting VEGF, PDGFR, FGFR or MET (e.g. nintedanib, vandetanib, sunitinib) [164] have been clinically evaluated, the survival outcome of these therapies are relatively limited and modest either by intrinsic or acquired resistance [165] or as monotherapy. These findings apply in particular to the treatment of NSCLC with a poor mean 5 year survival rate of less than 20% (REF). Patients with advanced or metastatic non-small cell lung cancer (NSCLC) without a treatable driver mutation who develop resistance to first-line checkpoint inhibitor (CPI) immunotherapy urgently need new second-line treatment options. Currently, only two anti-angiogenic compounds (nintedanib and ramucirumab) have been approved by the FDA and EMA for use in metastatic NSCLC without oncogenic driver mutations in combination with docetaxel after progression on chemotherapy [166, 167]. To this end, several combination strategies, including the combination of CPIs with anti-angiogenic tyrosine kinase inhibitors (VEGFR TKIs), are being tested in clinical trials (e.g. ramucirumab plus pembrolizumab) with promising preliminary results (REF). It has been hypothesized that anti-angiogenic treatment results in a vascular normalization. In principle, vessel normalization leads to a more homogeneous vessel density and thus to a more uniform perfusion of the flow bed [155] (Fig. 5). Morphologically, this effect is explained by a more stable connection of the endothelial cell assemblies, whose permeability is reduced by a uniform coverage with pericytes [168]. Under normal physiological conditions in the lung, a fragile balance of angiogenic and angiostatic factors regulates vascular homeostasis [169]. A causal explanation for vascular normalization in antiangiogenic therapy is seen in the blockade of the growth factor VEGF. This upregulates the molecule angiopoietin-1, which actively promotes the recruitment of additional pericytes and thus stabilizes the vessel wall in a supportive manner [170]. The normalization process of the tumor vasculature opens therapeutic opportunities to improve neoadjuvant chemotherapy and immunotherapy. The angio-immunogenic switch results in an improved perfusion and penetration of tumors by especially cytotoxic T lymphocytes (CTLs), effector T-cells, tumor-specific T-cell function, impaired T-cell memory, and a reduced immunosuppressive tumor microenvironment (TME). Compelling preclinical [171] and clinical [172, 173] evidence shows that targeting the VEGF and ANG2-Tie2-pathways seems to extend the transient window of vascular normalization and stimulates the intrinsically immunosuppressive tumor microenvironment (TME) in NSCLC and other solids tumors. Although the promising clinical evidence of an angio-immunogenic switch by combinational efficacy of anti-angiogenic agents and immune checkpoint inhibitors, it seems controversial that anti-angiogenic approaches in eligible patients with advanced NSCLC without oncogenic driver mutations are subject of debates despite the fundamental clinical breakthrough of immuno-oncology in the treatment of NSCLC (Fig. 5). This could be partly due to intrinsic and acquired resistance to anti-angiogenic treatments which have been particularly described in the clinical NSCLC trials as observed in the patient cohort of advanced, metastatic, EGFR-T790M-mutated patients [174]. Non-angiogenic mechanisms in lung cancer include vessel co-option (VC) [175, 176] and vasculogenic mimicry (VM) [177], both mechanisms making use (“hijacking”) of the preexisting vascularity. In specimens from early stage lung cancer, predominantly non-angiogenic mechanisms comprised 16% of all cases [178]. Moreover, in the mouse model extended sunitinib therapy is associated with VC as the predominant vascularization mechanism and ultimately treatment failure [176] (Table 2).

**Table 2:** Overview on mechanistic features of tumor angiogenesis

	Sprouting angiogenesis	Intussusceptive angiogenesis	Vessel mimicry	Vessel Co-option
Mechanism	Sprouting of endothelial cells from pre-existing vessels by proliferation and migration of endothelial cells	Existing blood vessels split into two new vessels by tissue pillars growing into the vessel lumen (vessel splitting)	Formation of vessel-like structures without true endothelial cells for the transport of blood	Hijacking of existing blood vessels by cancer cells or other cells to supply their growth. It does not involve the formation of new vessels or vessel remodeling
Key Molecules	Vascular Endothelial Growth Factor (VEGF), Fibroblast Growth Factor (FGF), Notch signaling	Angiopoietins, transforming growth factor-beta (TGF-beta), and extracellular matrix components	Matrix metalloproteinases (MMPs), VEGF and FGF, VE-Cadherin, EphA2, Nodal and HIF-1 α	Chemotactic signals, integrins, and extracellular matrix interactions are key components of vessel co-option
Characteristics	New vessels with tip cells guiding the sprout and stalk cells forming the main body of the new vessel	Division of existing vessels. It is important for vascular network remodeling	This process is often associated with aggressive tumors and contributes to their vascular supply	Rather than creating new vessels, cells use existing ones, often seen in non-angiogenic tumor growth

In many tumors, intussusceptive vascular growth is an escape mechanism that leads to resistance to many therapeutic regimens [179]. Especially after anti-angiogenic therapy or radiotherapy, there is often a switch from sprouting angiogenesis to intussusceptive expansion of tumor vessels [180]. While sprouting angiogenesis depends on the integrity of the VEGF signaling cascade, intussusceptive tumor angiogenesis usually follows several different stimuli such as intraluminal shear forces and perfusion differences within the tumor stromal area. In addition, an increase in the growth factor FGF-2 is observed, which allows the therapy-resistant tumor cells to continue to receive sufficient nutritive oxygen. Circulating endothelial progenitor cells have been postulated to be a critical component of tumor angiogenesis [25]. However, recruitment and incorporation of bone marrow-derived endothelial progenitor cells has been described in only about 5% of human tumor vessels [181]. However, in different mouse models, the proportion of incorporated progenitor cells varies considerably depending on the tumor entity.Similarly, circulating endothelial progenitor cells are thought to not only play a fundamental role in vascular differentiation, but also contribute in a paracrine manner to further vascular growth in tumor tissue through the release of proangiogenic growth factors. For example, the number of circulating endothelial progenitor cells positively correlates with tumor angiogenesis [182]. However, therapy with anti-angiogenic agents also significantly decreased the number of peripherally circulating endothelial progenitor cells [183]. Taken together, the combination of anti-angiogenic therapy with checkpoint inhibitors may potentiate the cancer immunity by normalizing the vascular architecture.

## Data Availability

No datasets were generated or analysed during the current study.

## References

[1] WHO. World Health Statistics 2014 Date last updated May 15 2014. Available from: https://www.who.int/news/item/15-05-2014-world-health-statistics-2014. Date last accessed: November 22 2023

[2] Hermanns MI, Unger RE, Kehe K et al (2004) Lung epithelial cell lines in coculture with human pulmonary microvascular endothelial cells: development of an alveolo-capillary barrier in vitro. Lab Invest 84(6):736–752 15077120 10.1038/labinvest.3700081

[3] Voswinckel R, Motejl V, Fehrenbach A et al (2004) Characterisation of post-pneumonectomy lung growth in adult mice. Eur Respir J 24(4):524–532 15459128 10.1183/09031936.04.10004904

[4] Ackermann M, Houdek JP, Gibney BC et al (2014) Sprouting and intussusceptive angiogenesis in postpneumonectomy lung growth: mechanisms of alveolar neovascularization. Angiogenesis 17(3):541–551 24150281 10.1007/s10456-013-9399-9 PMC4061467

[5] Butler JP, Loring SH, Patz S et al (2012) Evidence for adult lung growth in humans. N Engl J Med 367(3):244–247 22808959 10.1056/NEJMoa1203983 PMC3422892

[6] Kotton DN, Morrisey EE (2014) Lung regeneration: mechanisms, applications and emerging stem cell populations. Nat Med 20(8):822–832 25100528 10.1038/nm.3642 PMC4229034

[7] Basil MC, Katzen J, Engler AE et al (2020) The cellular and physiological basis for lung repair and regeneration: past, present, and future. Cell Stem Cell 26(4):482–502 32243808 10.1016/j.stem.2020.03.009 PMC7128675

[8] Mitzner W, Wagner EM (2004) Vascular remodeling in the circulations of the lung. J Appl Physiol 97(5):1999–2004 15475558 10.1152/japplphysiol.00473.2004

[9] Paredi P, Barnes PJ (2009) The airway vasculature: recent advances and clinical implications. Thorax 64(5):444–450 19401490 10.1136/thx.2008.100032

[10] Ackermann M, Tafforeau P, Wagner WL et al (2022) The bronchial circulation in COVID-19 Pneumonia. Am J Respir Crit Care Med 205(1):121–125 34734553 10.1164/rccm.202103-0594IM PMC8865596

[11] Weibel E (1959) Blood vessel anastomoses in the human lungs. Z Zellforsch Mikrosk Anat 50:653–692 13843453 10.1007/BF00381677

[12] Hayek Hv. Die menschliche Lunge Springer Berlin 2013.

[13] Ravnic DJ, Konerding MA, Pratt JP et al (2007) The murine bronchopulmonary microcirculation in hapten-induced inflammation. J Thorac Cardiovasc Surg 133(1):97–103 17198790 10.1016/j.jtcvs.2006.08.054

[14] Galambos C, Sims-Lucas S, Abman SH, Cool CD (2016) Intrapulmonary bronchopulmonary anastomoses and plexiform lesions in idiopathic pulmonary arterial hypertension. Am J Respir Crit Care Med 193(5):574–576 26930433 10.1164/rccm.201507-1508LE PMC4824924

[15] Galambos C, Bates ML, Bush D, Abman SH (2018) Prominent bronchopulmonary vascular anastomoses in fatal childhood asthma. Ann Am Thorac Soc 15(11):1359–1362 30079749 10.1513/AnnalsATS.201804-262RL PMC6322010

[16] Harbut P, Prisk GK, Lindwall R et al (2023) Intrapulmonary shunt and alveolar dead space in a cohort of patients with acute COVID-19 pneumonitis and early recovery. Eur Respir J 61(1):2201117 36137595 10.1183/13993003.01117-2022 PMC9515481

[17] Aird WC (2007) Phenotypic heterogeneity of the endothelium: II. Representative vascular beds Circ Res 100(2):174–190 17272819 10.1161/01.RES.0000255690.03436.ae

[18] Fuchs A, Weibel ER (1966) Morphometric study of the distribution of a specific cytoplasmatic organoid in the rat’s endothelial cells. Z Zellforsch Mikrosk Anat 73(1):1–9 5988362 10.1007/BF00348463

[19] Rondaij MG, Bierings R, Kragt A et al (2006) Dynamics and plasticity of Weibel-Palade bodies in endothelial cells. Arterioscler Thromb Vasc Biol 26(5):1002–1007 16469951 10.1161/01.ATV.0000209501.56852.6c

[20] Moldobaeva A, Wagner EM (2002) Heterogeneity of bronchial endothelial cell permeability. Am J Physiol Lung Cell Mol Physiol 283(3):L520–L527 12169570 10.1152/ajplung.00451.2001

[21] Schupp JC, Adams TS, Cosme C Jr et al (2021) Integrated single-cell atlas of endothelial cells of the human lung. Circulation 144(4):286–302 34030460 10.1161/CIRCULATIONAHA.120.052318 PMC8300155

[22] Chamoto K, Gibney BC, Ackermann M et al (2012) Alveolar macrophage dynamics in murine lung regeneration. J Cell Physiol 227(9):3208–3215 22105735 10.1002/jcp.24009 PMC4438671

[23] Chamoto K, Gibney BC, Lee GS et al (2013) Migration of CD11b+ accessory cells during murine lung regeneration. Stem Cell Res 10(3):267–277 23376466 10.1016/j.scr.2012.12.006 PMC3622126

[24] Ysasi AB, Wagner WL, Valenzuela CD et al (2017) Evidence for pleural epithelial-mesenchymal transition in murine compensatory lung growth. PLoS ONE 12(5):e0177921 28542402 10.1371/journal.pone.0177921 PMC5438137

[25] Mentzer SJ, Konerding MA (2014) Intussusceptive angiogenesis: expansion and remodeling of microvascular networks. Angiogenesis 17(3):499–509 24668225 10.1007/s10456-014-9428-3 PMC4063884

[26] Mentzer SJ, Ackermann M, Jonigk D (2022) Endothelialitis, microischemia, and intussusceptive angiogenesis in COVID-19. Cold Spring Harb Perspect Med. 10.1101/cshperspect.a041157 35534210 10.1101/cshperspect.a041157 PMC9524390

[27] Werlein C, Ackermann M, Stark H et al (2023) Inflammation and vascular remodeling in COVID-19 hearts. Angiogenesis 26(2):233–248 36371548 10.1007/s10456-022-09860-7 PMC9660162

[28] Peichev M, Naiyer AJ, Pereira D et al (2000) Expression of VEGFR-2 and AC133 by circulating human CD34(+) cells identifies a population of functional endothelial precursors. Blood 95(3):952–958 10648408 10.1182/blood.V95.3.952.003k27_952_958

[29] Belle J, Ysasi A, Bennett RD et al (2014) Stretch-induced intussuceptive and sprouting angiogenesis in the chick chorioallantoic membrane. Microvasc Res 95:60–67 24984292 10.1016/j.mvr.2014.06.009 PMC4188740

[30] Dimova I, Karthik S, Makanya A et al (2019) SDF-1/CXCR4 signalling is involved in blood vessel growth and remodelling by intussusception. J Cell Mol Med 23(6):3916–3926 30950188 10.1111/jcmm.14269 PMC6533523

[31] Patan S, Alvarez MJ, Schittny JC, Burri PH (1992) Intussusceptive microvascular growth: a common alternative to capillary sprouting. Arch Histol Cytol 55(Suppl):65–75 1290678 10.1679/aohc.55.Suppl_65

[32] Burri PH, Djonov V (2002) Intussusceptive angiogenesis–the alternative to capillary sprouting. Mol Aspects Med 23(6s):S1-27 12537983 10.1016/S0098-2997(02)00096-1

[33] Erba P, Ogawa R, Ackermann M et al (2011) Angiogenesis in wounds treated by microdeformational wound therapy. Ann Surg 253(2):402–409 21217515 10.1097/SLA.0b013e31820563a8 PMC3403722

[34] Ackermann M, Tsuda A, Secomb TW et al (2013) Intussusceptive remodeling of vascular branch angles in chemically-induced murine colitis. Microvasc Res 87:75–82 23485588 10.1016/j.mvr.2013.02.002 PMC3627825

[35] Ackermann M, Wagner WL, Rellecke P et al (2020) Parvovirus B19-induced angiogenesis in fulminant myocarditis. Eur Heart J 41(12):1309 32101607 10.1093/eurheartj/ehaa092

[36] Ackermann M, Morse BA, Delventhal V et al (2012) Anti-VEGFR2 and anti-IGF-1R-Adnectins inhibit Ewing’s sarcoma A673-xenograft growth and normalize tumor vascular architecture. Angiogenesis 15(4):685–695 22914877 10.1007/s10456-012-9294-9

[37] Ribatti D, Djonov V (2012) Intussusceptive microvascular growth in tumors. Cancer Lett 316(2):126–131 22197620 10.1016/j.canlet.2011.10.040

[38] Geudens I, Gerhardt H (2011) Coordinating cell behaviour during blood vessel formation. Development 138(21):4569–4583 21965610 10.1242/dev.062323

[39] Ribatti D, Crivellato E (2012) “Sprouting angiogenesis”, a reappraisal. Dev Biol 372(2):157–165 23031691 10.1016/j.ydbio.2012.09.018

[40] Jakobsson L, Franco CA, Bentley K et al (2010) Endothelial cells dynamically compete for the tip cell position during angiogenic sprouting. Nat Cell Biol 12(10):943–953 20871601 10.1038/ncb2103

[41] Eilken HM, Adams RH (2010) Dynamics of endothelial cell behavior in sprouting angiogenesis. Curr Opin Cell Biol 22(5):617–625 20817428 10.1016/j.ceb.2010.08.010

[42] Gomez-Salinero JM, Rafii S (2018) Endothelial cell adaptation in regeneration. Science 362(6419):1116–1117 30523098 10.1126/science.aar4800

[43] Psaltis PJ, Simari RD (2015) Vascular wall progenitor cells in health and disease. Circ Res 116(8):1392–1412 25858065 10.1161/CIRCRESAHA.116.305368

[44] Medina RJ, Smadja DM (2019) Editorial: recent advances in endothelial progenitor cells toward their use in clinical translation. Front Med (Lausanne) 6:290 31970157 10.3389/fmed.2019.00290 PMC6960121

[45] Smadja DM (2019) Vasculogenic Stem and progenitor cells in human: future cell therapy product or liquid biopsy for vascular disease. Adv Exp Med Biol 1201:215–237 31898789 10.1007/978-3-030-31206-0_11

[46] Asahara T, Murohara T, Sullivan A et al (1997) Isolation of putative progenitor endothelial cells for angiogenesis. Science 275(5302):964–967 9020076 10.1126/science.275.5302.964

[47] Yoder MC, Mead LE, Prater D et al (2007) Redefining endothelial progenitor cells via clonal analysis and hematopoietic stem/progenitor cell principals. Blood 109(5):1801–1809 17053059 10.1182/blood-2006-08-043471 PMC1801067

[48] Ng CY, Cheung C (2024) Origins and functional differences of blood endothelial cells. Semin Cell Dev Biol 155(Pt C):23–29 37202277 10.1016/j.semcdb.2023.05.001

[49] Smadja DM, Dorfmüller P, Guerin CL et al (2014) Cooperation between human fibrocytes and endothelial colony-forming cells increases angiogenesis via the CXCR4 pathway. Thromb Haemost 112(5):1002–1013 25103869 10.1160/TH13-08-0711 PMC4751883

[50] Dragoni S, Laforenza U, Bonetti E et al (2011) Vascular endothelial growth factor stimulates endothelial colony forming cells proliferation and tubulogenesis by inducing oscillations in intracellular Ca2+ concentration. Stem Cells 29(11):1898–1907 21905169 10.1002/stem.734

[51] Evrard SM, d’Audigier C, Mauge L et al (2012) The profibrotic cytokine transforming growth factor-β1 increases endothelial progenitor cell angiogenic properties. J Thromb Haemost 10(4):670–679 22284809 10.1111/j.1538-7836.2012.04644.x

[52] Smadja DM, Rossi E, Haviari S et al (2023) Thrombin receptor PAR1 silencing in endothelial colony-forming cells modifies stemness and vasculogenic properties. J Thromb Haemost 21(12):3640–3648 37678550 10.1016/j.jtha.2023.08.029

[53] Cras A, Larghero J, Rossi E et al (2023) Nestin is a new partner in endothelial colony forming cell angiogenic potential. Stem Cell Rev Rep 19(7):2541–2550 37452965 10.1007/s12015-023-10587-1

[54] Benslimane-Ahmim Z, Heymann D, Dizier B et al (2011) Osteoprotegerin, a new actor in vasculogenesis, stimulates endothelial colony-forming cells properties. J Thromb Haemost 9(4):834–843 21255246 10.1111/j.1538-7836.2011.04207.x

[55] Gouzi F, Philippe A, Blervaque L et al (2022) Plasma ratio of angiopoietin-2 to angiopoietin-1 is a biomarker of vascular impairment in chronic obstructive pulmonary disease patients. Angiogenesis 25(3):275–277 35013842 10.1007/s10456-021-09826-1

[56] Smadja DM, Mauge L, Nunes H et al (2013) Imbalance of circulating endothelial cells and progenitors in idiopathic pulmonary fibrosis. Angiogenesis 16(1):147–157 22983452 10.1007/s10456-012-9306-9

[57] Billoir P, Blandinières A, Gendron N et al (2021) Endothelial colony-forming cells from idiopathic pulmonary fibrosis patients have a high procoagulant potential. Stem Cell Rev Rep 17(2):694–699 32970229 10.1007/s12015-020-10043-4

[58] Paschalaki KE, Randi AM (2018) Recent advances in endothelial colony forming cells toward their use in clinical translation. Front Med (Lausanne) 5:295 30406106 10.3389/fmed.2018.00295 PMC6205967

[59] Blandinières A, Gille T, Sadoine J et al (2018) Endothelial colony-forming cells do not participate to fibrogenesis in a bleomycin-induced pulmonary fibrosis model in nude mice. Stem Cell Rev Rep 14(6):812–822 30267203 10.1007/s12015-018-9846-5

[60] Paschalaki K, Rossios C, Pericleous C et al (2022) Inhaled corticosteroids reduce senescence in endothelial progenitor cells from patients with COPD. Thorax 77(6):616–620 35027472 10.1136/thoraxjnl-2020-216807 PMC9120381

[61] Blervaque L, Pomiès P, Rossi E et al (2020) COPD is deleterious for pericytes: implications during training-induced angiogenesis in skeletal muscle. Am J Physiol Heart Circ Physiol 319(5):H1142–H1151 32986960 10.1152/ajpheart.00306.2020

[62] Blandinières A, Gendron N, Bacha N et al (2019) Interleukin-8 release by endothelial colony-forming cells isolated from idiopathic pulmonary fibrosis patients might contribute to their pathogenicity. Angiogenesis 22(2):325–339 30607696 10.1007/s10456-018-09659-5

[63] Benyamine A, Magalon J, Cointe S et al (2017) Increased serum levels of fractalkine and mobilisation of CD34(+)CD45(-) endothelial progenitor cells in systemic sclerosis. Arthritis Res Ther 19(1):60 28320472 10.1186/s13075-017-1271-7 PMC5359964

[64] Duong HT, Comhair SA, Aldred MA et al (2011) Pulmonary artery endothelium resident endothelial colony-forming cells in pulmonary arterial hypertension. Pulm Circ 1(4):475–486 22530103 10.4103/2045-8932.93547 PMC3329078

[65] Toshner M, Voswinckel R, Southwood M et al (2009) Evidence of dysfunction of endothelial progenitors in pulmonary arterial hypertension. Am J Respir Crit Care Med 180(8):780–787 19628780 10.1164/rccm.200810-1662OC PMC2778151

[66] Smadja DM, Levy M, Huang L et al (2015) Treprostinil indirectly regulates endothelial colony forming cell angiogenic properties by increasing VEGF-A produced by mesenchymal stem cells. Thromb Haemost 114(4):735–747 26062754 10.1160/TH14-11-0907 PMC6570989

[67] Safiri S, Mahmoodpoor A, Kolahi AA et al (2022) Global burden of lower respiratory infections during the last three decades. Front Public Health 10:1028525 36699876 10.3389/fpubh.2022.1028525 PMC9869262

[68] Global, regional, and national age-sex-specific mortality for 282 causes of death in 195 countries and territories, 1980–2017: a systematic analysis for the Global Burden of Disease Study 2017. Lancet. 2018;392(10159):1736–88. 10.1016/S0140-6736(18)32203-7 PMC6227606 30496103

[69] Proud D, Leigh R (2011) Epithelial cells and airway diseases. Immunol Rev 242(1):186–204 21682746 10.1111/j.1600-065X.2011.01033.x

[70] Lubkin A, Torres VJ (2017) Bacteria and endothelial cells: a toxic relationship. Curr Opin Microbiol 35:58–63 28013162 10.1016/j.mib.2016.11.008 PMC5481510

[71] Aird WC (2007) Phenotypic heterogeneity of the endothelium: I. Structure, function, and mechanisms. Circ Res 100(2):158–173 17272818 10.1161/01.RES.0000255691.76142.4a

[72] Drakopanagiotakis F, Polychronopoulos V, Judson MA (2008) Organizing pneumonia. Am J Med Sci 335(1):34–39 18195581 10.1097/MAJ.0b013e31815d829d

[73] Cabrera-Benitez NE, Laffey JG, Parotto M et al (2014) Mechanical ventilation-associated lung fibrosis in acute respiratory distress syndrome: a significant contributor to poor outcome. Anesthesiology 121(1):189–198 24732023 10.1097/ALN.0000000000000264 PMC4991945

[74] Yamada M, Kubo H, Ishizawa K et al (2005) Increased circulating endothelial progenitor cells in patients with bacterial pneumonia: evidence that bone marrow derived cells contribute to lung repair. Thorax 60(5):410–413 15860717 10.1136/thx.2004.034058 PMC1758906

[75] Ackermann M, Stark H, Neubert L et al (2020) Morphomolecular motifs of pulmonary neoangiogenesis in interstitial lung diseases. Eur Respir J. 10.1183/13993003.00933-2019 31806721 10.1183/13993003.00933-2019

[76] Rafat N, Tönshoff B, Bierhaus A, Beck GC (2013) Endothelial progenitor cells in regeneration after acute lung injury: do they play a role? Am J Respir Cell Mol Biol 48(4):399–405 23103996 10.1165/rcmb.2011-0132TR

[77] Chamoto K, Gibney BC, Lee GS et al (2012) CD34+ progenitor to endothelial cell transition in post-pneumonectomy angiogenesis. Am J Respir Cell Mol Biol 46(3):283–289 21921238 10.1165/rcmb.2011-0249OC PMC3326434

[78] Schniedermann J, Rennecke M, Buttler K et al (2010) Mouse lung contains endothelial progenitors with high capacity to form blood and lymphatic vessels. BMC Cell Biol 11:50 20594323 10.1186/1471-2121-11-50 PMC2911414

[79] Armstrong SM, Wang C, Tigdi J et al (2012) Influenza infects lung microvascular endothelium leading to microvascular leak: role of apoptosis and claudin-5. PLoS ONE 7(10):e47323 23115643 10.1371/journal.pone.0047323 PMC3480371

[80] Ogiwara H, Yasui F, Munekata K et al (2014) Histopathological evaluation of the diversity of cells susceptible to H5N1 virulent avian influenza virus. Am J Pathol 184(1):171–183 24200852 10.1016/j.ajpath.2013.10.004 PMC3873492

[81] Rommel MGE, Milde C, Eberle R et al (2020) Endothelial-platelet interactions in influenza-induced pneumonia: A potential therapeutic target. Anat Histol Embryol 49(5):606–619 31793053 10.1111/ahe.12521

[82] Sugiyama MG, Gamage A, Zyla R et al (2016) Influenza virus infection induces platelet-endothelial adhesion which contributes to lung injury. J Virol 90(4):1812–1823 26637453 10.1128/JVI.02599-15 PMC4733979

[83] Wang C, Armstrong SM, Sugiyama MG et al (2015) Influenza-induced priming and leak of human lung microvascular endothelium upon Exposure to Staphylococcus aureus. Am J Respir Cell Mol Biol 53(4):459–470 25693001 10.1165/rcmb.2014-0373OC

[84] Ackermann M, Verleden SE, Kuehnel M et al (2020) Pulmonary vascular endothelialitis, thrombosis, and angiogenesis in Covid-19. N Engl J Med 383(2):120–128 32437596 10.1056/NEJMoa2015432 PMC7412750

[85] Ackermann M, Anders HJ, Bilyy R et al (2021) Patients with COVID-19: in the dark-NETs of neutrophils. Cell Death Differ 28(11):3125–3139 34031543 10.1038/s41418-021-00805-z PMC8142290

[86] Dhont S, Derom E, Van Braeckel E et al (2020) The pathophysiology of “happy” hypoxemia in COVID-19. Respir Res 21(1):198 32723327 10.1186/s12931-020-01462-5 PMC7385717

[87] Loring SH, Garcia-Jacques M, Malhotra A (2009) Pulmonary characteristics in COPD and mechanisms of increased work of breathing. J Appl Physiol 107(1):309–314 19359620 10.1152/japplphysiol.00008.2009 PMC2711781

[88] Liu F, Han K, Blair R et al (2021) SARS-CoV-2 infects endothelial cells in vivo and in vitro. Front Cell Infect Microbiol 11:701278 34307198 10.3389/fcimb.2021.701278 PMC8292147

[89] Cardot-Leccia N, Hubiche T, Dellamonica J et al (2020) Pericyte alteration sheds light on micro-vasculopathy in COVID-19 infection. Intensive Care Med 46(9):1777–1778 32533198 10.1007/s00134-020-06147-7 PMC7291173

[90] Huertas A, Montani D, Savale L et al (2020) Endothelial cell dysfunction: a major player in SARS-CoV-2 infection (COVID-19)? Eur Respir J. 10.1183/13993003.01634-2020 32554538 10.1183/13993003.01634-2020 PMC7301835

[91] Baile EM (1996) The anatomy and physiology of the bronchial circulation. J Aerosol Med 9(1):1–6 10160199 10.1089/jam.1996.9.1

[92] Kroshus TJ, Kshettry VR, Hertz MI, Bolman RM 3rd (1995) Deep venous thrombosis and pulmonary embolism after lung transplantation. J Thorac Cardiovasc Surg 110(2):540–544 7637373 10.1016/S0022-5223(95)70252-0

[93] Walsh CL, Tafforeau P, Wagner WL et al (2021) Imaging intact human organs with local resolution of cellular structures using hierarchical phase-contrast tomography. Nat Methods 18(12):1532–1541 34737453 10.1038/s41592-021-01317-x PMC8648561

[94] Cervia-Hasler C, Brüningk SC, Hoch T et al (2024) Persistent complement dysregulation with signs of thromboinflammation in active Long Covid. Science. 10.1126/science.adg7942 38236961 10.1126/science.adg7942

[95] Konopka KE, Perry W, Huang T et al (2021) Usual interstitial pneumonia is the most common finding in surgical lung biopsies from patients with persistent interstitial lung disease following infection with SARS-CoV-2. EClinicalMedicine 42:101209 34841234 10.1016/j.eclinm.2021.101209 PMC8609167

[96] Ackermann M, Kamp JC, Werlein C et al (2022) The fatal trajectory of pulmonary COVID-19 is driven by lobular ischemia and fibrotic remodelling. EBioMedicine 85:104296 36206625 10.1016/j.ebiom.2022.104296 PMC9535314

[97] Smadja DM, Mentzer SJ, Fontenay M et al (2021) COVID-19 is a systemic vascular hemopathy: insight for mechanistic and clinical aspects. Angiogenesis 24(4):755–788 34184164 10.1007/s10456-021-09805-6 PMC8238037

[98] Smadja DM, Jannot AS, Philippe A et al (2023) Circulating Von Willebrand factor: a consistent biomarker predicting in-hospital mortality across different waves of the COVID-19 pandemic. Angiogenesis 27:1–4 38070063 10.1007/s10456-023-09901-9

[99] Chapuis N, Ibrahimi N, Belmondo T et al (2022) Dynamics of circulating calprotectin accurately predict the outcome of moderate COVID-19 patients. EBioMedicine 80:104077 35644124 10.1016/j.ebiom.2022.104077 PMC9132728

[100] Smadja DM, Gendron N, Philippe A et al (2023) Fibrin monomers evaluation during hospitalization for COVID-19 is a predictive marker of in-hospital mortality. Front Cardiovasc Med 10:1001530 37063947 10.3389/fcvm.2023.1001530 PMC10098364

[101] Philippe A, Günther S, Rancic J et al (2023) VEGF-A plasma levels are associated with impaired DLCO and radiological sequelae in long COVID patients. Angiogenesis 27:51–66 37526809 10.1007/s10456-023-09890-9

[102] Patel MA, Knauer MJ, Nicholson M et al (2022) Elevated vascular transformation blood biomarkers in Long-COVID indicate angiogenesis as a key pathophysiological mechanism. Mol Med 28(1):122 36217108 10.1186/s10020-022-00548-8 PMC9549814

[103] Smadja DM, Guerin CL, Chocron R et al (2020) Angiopoietin-2 as a marker of endothelial activation is a good predictor factor for intensive care unit admission of COVID-19 patients. Angiogenesis 23(4):611–620 32458111 10.1007/s10456-020-09730-0 PMC7250589

[104] Kovacs G, Agusti A, Barberà JA et al (2018) Pulmonary vascular involvement in chronic obstructive pulmonary disease. Is there a pulmonary vascular phenotype? Am J Respir Crit Care Med 198(8):1000–1011 29746142 10.1164/rccm.201801-0095PP

[105] Peinado VI, Barbera JA, Ramirez J et al (1998) Endothelial dysfunction in pulmonary arteries of patients with mild COPD. Am J Physiol 274(6):L908–L913 9609729 10.1152/ajplung.1998.274.6.L908

[106] Wiebe BM, Laursen H (1998) Lung morphometry by unbiased methods in emphysema: bronchial and blood vessel volume, alveolar surface area and capillary length. APMIS 106(6):651–656 9725798 10.1111/j.1699-0463.1998.tb01395.x

[107] Kropski JA, Richmond BW, Gaskill CF et al (2018) Deregulated angiogenesis in chronic lung diseases: a possible role for lung mesenchymal progenitor cells (2017 Grover Conference Series). Pulm Circ 8(1):2045893217739807 29040010 10.1177/2045893217739807 PMC5731726

[108] Hueper K, Vogel-Claussen J, Parikh MA et al (2015) Pulmonary microvascular blood flow in mild chronic obstructive pulmonary disease and emphysema. The MESA COPD study. Am J Respir Crit Care Med 192(5):570–580 26067761 10.1164/rccm.201411-2120OC PMC4595687

[109] Barker AL, Eddy RL, MacNeil JL et al (2021) CT pulmonary vessels and MRI ventilation in chronic obstructive pulmonary disease: Relationship with worsening FEV(1) in the TINCan cohort study. Acad Radiol 28(4):495–506 32303446 10.1016/j.acra.2020.03.006

[110] Hisata S, Racanelli AC, Kermani P et al (2021) Reversal of emphysema by restoration of pulmonary endothelial cells. J Exp Med. 10.1084/jem.20200938 34287647 10.1084/jem.20200938 PMC8298104

[111] Sauler M, McDonough JE, Adams TS et al (2022) Characterization of the COPD alveolar niche using single-cell RNA sequencing. Nat Commun 13(1):494 35078977 10.1038/s41467-022-28062-9 PMC8789871

[112] Wang L, Xu Z, Chen B et al (2017) The role of vascular endothelial growth factor in small-airway remodelling in a rat model of chronic obstructive pulmonary disease. Sci Rep 7:41202 28117425 10.1038/srep41202 PMC5259712

[113] Liebow AA (1959) Pulmonary emphysema with special reference to vascular changes. Am Rev Respir Dis 80:67–93 13670406 10.1164/arrd.1959.80.1P2.67

[114] Ono S, Westcott JY, Voelkel NF (1992) PAF antagonists inhibit pulmonary vascular remodeling induced by hypobaric hypoxia in rats. J Appl Physiol 73(3):1084–1092 1400021 10.1152/jappl.1992.73.3.1084

[115] Kasahara Y, Tuder RM, Cool CD et al (2001) Endothelial cell death and decreased expression of vascular endothelial growth factor and vascular endothelial growth factor receptor 2 in emphysema. Am J Respir Crit Care Med 163(3 Pt 1):737–744 11254533 10.1164/ajrccm.163.3.2002117

[116] Dinh-Xuan AT, Higenbottam TW, Clelland CA et al (1991) Impairment of endothelium-dependent pulmonary-artery relaxation in chronic obstructive lung disease. N Engl J Med 324(22):1539–1547 2027358 10.1056/NEJM199105303242203

[117] Daley E, Emson C, Guignabert C et al (2008) Pulmonary arterial remodeling induced by a Th2 immune response. J Exp Med 205(2):361–372 18227220 10.1084/jem.20071008 PMC2271018

[118] Yamaji-Kegan K, Takimoto E, Zhang A et al (2014) Hypoxia-induced mitogenic factor (FIZZ1/RELMα) induces endothelial cell apoptosis and subsequent interleukin-4-dependent pulmonary hypertension. Am J Physiol Lung Cell Mol Physiol 306(12):L1090–L1103 24793164 10.1152/ajplung.00279.2013 PMC4060011

[119] Sullivan AK, Simonian PL, Falta MT et al (2005) Oligoclonal CD4+ T cells in the lungs of patients with severe emphysema. Am J Respir Crit Care Med 172(5):590–596 15937291 10.1164/rccm.200410-1332OC PMC2718531

[120] Taraseviciene-Stewart L, Scerbavicius R, Choe KH et al (2005) An animal model of autoimmune emphysema. Am J Respir Crit Care Med 171(7):734–742 15563631 10.1164/rccm.200409-1275OC

[121] Humbert M, Guignabert C, Bonnet S et al (2019) Pathology and pathobiology of pulmonary hypertension: state of the art and research perspectives. Eur Respir J. 10.1183/13993003.01887-2018 30545970 10.1183/13993003.01887-2018 PMC6351340

[122] Jonigk D, Golpon H, Bockmeyer CL et al (2011) Plexiform lesions in pulmonary arterial hypertension composition, architecture, and microenvironment. Am J Pathol 179(1):167–179 21703400 10.1016/j.ajpath.2011.03.040 PMC3123793

[123] Westöö C, Norvik C, Peruzzi N et al (2021) Distinct types of plexiform lesions identified by synchrotron-based phase-contrast micro-CT. Am J Physiol Lung Cell Mol Physiol 321(1):L17-l28 33881927 10.1152/ajplung.00432.2020 PMC8321861

[124] Tio D, Leter E, Boerrigter B et al (2013) Risk factors for hemoptysis in idiopathic and hereditary pulmonary arterial hypertension. PLoS ONE 8(10):e78132 24194909 10.1371/journal.pone.0078132 PMC3806771

[125] Montani D, Lau EM, Dorfmüller P et al (2016) Pulmonary veno-occlusive disease. Eur Respir J 47(5):1518–1534 27009171 10.1183/13993003.00026-2016

[126] Weatherald J, Dorfmüller P, Perros F et al (2020) Pulmonary capillary haemangiomatosis: a distinct entity. Eur Respir Rev. 10.1183/16000617.0168-2019 32461209 10.1183/16000617.0168-2019 PMC9488541

[127] Neubert L, Borchert P, Shin HO et al (2019) Comprehensive three-dimensional morphology of neoangiogenesis in pulmonary veno-occlusive disease and pulmonary capillary hemangiomatosis. J Pathol Clin Res 5(2):108–114 30697960 10.1002/cjp2.125 PMC6463863

[128] Simonneau G, Torbicki A, Dorfmüller P, Kim N (2017) The pathophysiology of chronic thromboembolic pulmonary hypertension. Eur Respir Rev. 10.1183/16000617.0112-2016 28356405 10.1183/16000617.0112-2016 PMC9488693

[129] Ackermann M, Gaumann A, Mentzer SJ et al (2017) Plexiform vasculopathy in chronic thromboembolic pulmonary hypertension. Am J Respir Crit Care Med 196(8):e48–e51 28892403 10.1164/rccm.201703-0591IM

[130] Lang IM, Dorfmüller P, Vonk NA (2016) The pathobiology of chronic thromboembolic pulmonary hypertension. Ann Am Thorac Soc 13(Suppl 3):S215–S221 27571003 10.1513/AnnalsATS.201509-620AS

[131] Jonigk D, Stark H, Braubach P et al (2019) Morphological and molecular motifs of fibrosing pulmonary injury patterns. J Pathol Clin Res 5(4):256–271 31433553 10.1002/cjp2.141 PMC6817833

[132] Puxeddu E, Cavalli F, Pezzuto G et al (2017) Impact of pulmonary vascular volume on mortality in IPF: is it time to reconsider the role of vasculature in disease pathogenesis and progression? Eur Respir J. 10.1183/13993003.02345-2016 28232417 10.1183/13993003.02345-2016

[133] Colombat M, Mal H, Groussard O et al (2007) Pulmonary vascular lesions in end-stage idiopathic pulmonary fibrosis: Histopathologic study on lung explant specimens and correlations with pulmonary hemodynamics. Hum Pathol 38(1):60–65 16949908 10.1016/j.humpath.2006.06.007

[134] Caporarello N, Ligresti G (2023) Vascular contribution to lung repair and fibrosis. Am J Respir Cell Mol Biol 69(2):135–146 37126595 10.1165/rcmb.2022-0431TR PMC10399144

[135] Probst CK, Montesi SB, Medoff BD et al (2020) Vascular permeability in the fibrotic lung. Eur Respir J. 10.1183/13993003.00100-2019 32265308 10.1183/13993003.00100-2019 PMC9977144

[136] Leach HG, Chrobak I, Han R, Trojanowska M (2013) Endothelial cells recruit macrophages and contribute to a fibrotic milieu in bleomycin lung injury. Am J Respir Cell Mol Biol 49(6):1093–1101 23885794 10.1165/rcmb.2013-0152OC PMC3931119

[137] Sriram K, Laughlin JG, Rangamani P, Tartakovsky DM (2016) Shear-induced nitric oxide production by endothelial cells. Biophys J 111(1):208–221 27410748 10.1016/j.bpj.2016.05.034 PMC4944664

[138] Sung ML, Wu CC, Chang HI et al (2009) Shear stress inhibits homocysteine-induced stromal cell-derived factor-1 expression in endothelial cells. Circ Res 105(8):755–763 19745163 10.1161/CIRCRESAHA.109.206524 PMC2771228

[139] Negri S, Faris P, Berra-Romani R et al (2019) Endothelial transient receptor potential channels and vascular remodeling: extracellular Ca(2 +) entry for angiogenesis, arteriogenesis and vasculogenesis. Front Physiol 10:1618 32038296 10.3389/fphys.2019.01618 PMC6985578

[140] Kamp JC, Neubert L, Ackermann M et al (2022) A morphomolecular approach to alveolar capillary dysplasia. Am J Pathol 192(8):1110–1121 35649494 10.1016/j.ajpath.2022.05.004

[141] Ebina M, Shimizukawa M, Shibata N et al (2004) Heterogeneous increase in CD34-positive alveolar capillaries in idiopathic pulmonary fibrosis. Am J Respir Crit Care Med 169(11):1203–1208 14754760 10.1164/rccm.200308-1111OC

[142] Lapenna A, De Palma M, Lewis CE (2018) Perivascular macrophages in health and disease. Nat Rev Immunol 18(11):689–702 30127389 10.1038/s41577-018-0056-9

[143] Rossi G, Cavazza A, Spagnolo P et al (2017) The role of macrophages in interstitial lung diseases: Number 3 in the Series “Pathology for the clinician” Edited by Peter Dorfmüller and Alberto Cavazza. Eur Respir Rev. 10.1183/16000617.0009-2017 28724562 10.1183/16000617.0009-2017 PMC9488916

[144] Keane MP, Strieter RM, Lynch JP 3rd, Belperio JA (2006) Inflammation and angiogenesis in fibrotic lung disease. Semin Respir Crit Care Med 27(6):589–599 17195136 10.1055/s-2006-957331

[145] Schupp JC, Manning EP, Chioccioli M et al (2023) Alveolar vascular remodeling in nonspecific interstitial pneumonia: replacement of normal lung capillaries with COL15A1-positive endothelial cells. Am J Respir Crit Care Med 208(7):819–822 37552025 10.1164/rccm.202303-0544LE PMC10563189

[146] Adams TS, Schupp JC, Poli S et al (2020) Single-cell RNA-seq reveals ectopic and aberrant lung-resident cell populations in idiopathic pulmonary fibrosis. Sci Adv. 10.1126/sciadv.aba1983 32832599 10.1126/sciadv.aba1983 PMC7439502

[147] Turner-Warwick M (1963) Precapillary systemic-pulmonary anastomoses. Thorax 18(3):225–237 14064617 10.1136/thx.18.3.225 PMC1018768

[148] Hanumegowda C, Farkas L, Kolb M (2012) Angiogenesis in pulmonary fibrosis: too much or not enough? Chest 142(1):200–207 22796840 10.1378/chest.11-1962

[149] Cao Z, Lis R, Ginsberg M et al (2016) Targeting of the pulmonary capillary vascular niche promotes lung alveolar repair and ameliorates fibrosis. Nat Med 22(2):154–162 26779814 10.1038/nm.4035 PMC4872630

[150] Jia W, Wang Z, Gao C et al (2021) Trajectory modeling of endothelial-to-mesenchymal transition reveals galectin-3 as a mediator in pulmonary fibrosis. Cell Death Dis 12(4):327 33771973 10.1038/s41419-021-03603-0 PMC7998015

[151] Richeldi L, du Bois RM, Raghu G et al (2014) Efficacy and safety of nintedanib in idiopathic pulmonary fibrosis. N Engl J Med 370(22):2071–2082 24836310 10.1056/NEJMoa1402584

[152] Wollin L, Distler JHW, Redente EF et al (2019) Potential of nintedanib in treatment of progressive fibrosing interstitial lung diseases. Eur Respir J. 10.1183/13993003.00161-2019 31285305 10.1183/13993003.00161-2019 PMC6751387

[153] Distler O, Highland KB, Gahlemann M et al (2019) Nintedanib for systemic sclerosis-associated interstitial lung disease. N Engl J Med 380(26):2518–2528 31112379 10.1056/NEJMoa1903076

[154] Ackermann M, Kim YO, Wagner WL et al (2017) Effects of nintedanib on the microvascular architecture in a lung fibrosis model. Angiogenesis 20(3):359–372 28283856 10.1007/s10456-017-9543-z PMC7412751

[155] Jain RK (2005) Normalization of tumor vasculature: an emerging concept in antiangiogenic therapy. Science 307(5706):58–62 15637262 10.1126/science.1104819

[156] Folkman J (1971) Tumor angiogenesis: therapeutic implications. N Engl J Med 285(21):1182–1186 4938153 10.1056/NEJM197111182852108

[157] Bergers G, Benjamin LE (2003) Tumorigenesis and the angiogenic switch. Nat Rev Cancer 3(6):401–410 12778130 10.1038/nrc1093

[158] Konerding MA, Malkusch W, Klapthor B et al (1999) Evidence for characteristic vascular patterns in solid tumours: quantitative studies using corrosion casts. Br J Cancer 80(5–6):724–732 10360650 10.1038/sj.bjc.6690416 PMC2362271

[159] Morikawa S, Baluk P, Kaidoh T et al (2002) Abnormalities in pericytes on blood vessels and endothelial sprouts in tumors. Am J Pathol 160(3):985–1000 11891196 10.1016/S0002-9440(10)64920-6 PMC1867175

[160] Heldin CH, Rubin K, Pietras K, Ostman A (2004) High interstitial fluid pressure—an obstacle in cancer therapy. Nat Rev Cancer 4(10):806–813 15510161 10.1038/nrc1456

[161] McDonald DM, Choyke PL (2003) Imaging of angiogenesis: from microscope to clinic. Nat Med 9(6):713–725 12778170 10.1038/nm0603-713

[162] Konerding MA, Fait E, Gaumann A (2001) 3D microvascular architecture of pre-cancerous lesions and invasive carcinomas of the colon. Br J Cancer 84(10):1354–1362 11355947 10.1054/bjoc.2001.1809 PMC2363651

[163] Eldridge L, Wagner EM (2019) Angiogenesis in the lung. J Physiol 597(4):1023–1032 30022479 10.1113/JP275860 PMC6376070

[164] Crinò L, Metro G (2014) Therapeutic options targeting angiogenesis in nonsmall cell lung cancer. Eur Respir Rev 23(131):79–91 24591665 10.1183/09059180.00008913 PMC9487252

[165] Eelen G, Treps L, Li X, Carmeliet P (2020) Basic and therapeutic aspects of angiogenesis updated. Circ Res 127(2):310–329 32833569 10.1161/CIRCRESAHA.120.316851

[166] Popat S, Mellemgaard A, Fahrbach K et al (2015) Nintedanib plus docetaxel as second-line therapy in patients with non-small-cell lung cancer: a network meta-analysis. Future Oncol 11(3):409–420 25478720 10.2217/fon.14.290

[167] Larkins E, Scepura B, Blumenthal GM et al (2015) U.S. food and drug administration approval summary: ramucirumab for the treatment of metastatic non-small cell lung cancer following disease progression on or after platinum-based chemotherapy. Oncologist 20(11):1320–1325 26446239 10.1634/theoncologist.2015-0221 PMC4718430

[168] Goel S, Duda DG, Xu L et al (2011) Normalization of the vasculature for treatment of cancer and other diseases. Physiol Rev 91(3):1071–1121 21742796 10.1152/physrev.00038.2010 PMC3258432

[169] Fukumura D, Kloepper J, Amoozgar Z et al (2018) Enhancing cancer immunotherapy using antiangiogenics: opportunities and challenges. Nat Rev Clin Oncol 15(5):325–340 29508855 10.1038/nrclinonc.2018.29 PMC5921900

[170] Carmeliet P, Jain RK (2011) Principles and mechanisms of vessel normalization for cancer and other angiogenic diseases. Nat Rev Drug Discov 10(6):417–427 21629292 10.1038/nrd3455

[171] Allen E, Jabouille A, Rivera LB et al (2017) Combined antiangiogenic and anti-PD-L1 therapy stimulates tumor immunity through HEV formation. Sci Transl Med. 10.1126/scitranslmed.aak9679 28404866 10.1126/scitranslmed.aak9679 PMC5554432

[172] Le Tourneau C, Becker H, Claus R et al (2022) Two phase I studies of BI 836880, a vascular endothelial growth factor/angiopoietin-2 inhibitor, administered once every 3 weeks or once weekly in patients with advanced solid tumors. ESMO Open 7(5):100576 36108560 10.1016/j.esmoop.2022.100576 PMC9588896

[173] Khan KA, Wu FT, Cruz-Munoz W, Kerbel RS (2021) Ang2 inhibitors and Tie2 activators: potential therapeutics in perioperative treatment of early stage cancer. EMBO Mol Med 13(7):e08253 34125494 10.15252/emmm.201708253 PMC8261516

[174] Kawashima Y, Fukuhara T, Saito H et al (2022) Bevacizumab plus erlotinib versus erlotinib alone in Japanese patients with advanced, metastatic, EGFR-mutant non-small-cell lung cancer (NEJ026): overall survival analysis of an open-label, randomised, multicentre, phase 3 trial. Lancet Respir Med 10(1):72–82 34454653 10.1016/S2213-2600(21)00166-1

[175] Kuczynski EA, Vermeulen PB, Pezzella F et al (2019) Vessel co-option in cancer. Nat Rev Clin Oncol 16(8):469–493 30816337 10.1038/s41571-019-0181-9

[176] Teuwen LA, De Rooij L, Cuypers A et al (2021) Tumor vessel co-option probed by single-cell analysis. Cell Rep 35(11):109253 34133923 10.1016/j.celrep.2021.109253

[177] Maniotis AJ, Folberg R, Hess A et al (1999) Vascular channel formation by human melanoma cells in vivo and in vitro: vasculogenic mimicry. Am J Pathol 155(3):739–752 10487832 10.1016/S0002-9440(10)65173-5 PMC1866899

[178] Pezzella F, Pastorino U, Tagliabue E et al (1997) Non-small-cell lung carcinoma tumor growth without morphological evidence of neo-angiogenesis. Am J Pathol 151(5):1417–1423 9358768 PMC1858069

[179] Hlushchuk R, Ehrbar M, Reichmuth P et al (2011) Decrease in VEGF expression induces intussusceptive vascular pruning. Arterioscler Thromb Vasc Biol 31(12):2836–2844 21921259 10.1161/ATVBAHA.111.231811

[180] Hlushchuk R, Riesterer O, Baum O et al (2008) Tumor recovery by angiogenic switch from sprouting to intussusceptive angiogenesis after treatment with PTK787/ZK222584 or ionizing radiation. Am J Pathol 173(4):1173–1185 18787105 10.2353/ajpath.2008.071131 PMC2543084

[181] Peters BA, Diaz LA, Polyak K et al (2005) Contribution of bone marrow-derived endothelial cells to human tumor vasculature. Nat Med 11(3):261–262 15723071 10.1038/nm1200

[182] Bertolini F, Paul S, Mancuso P et al (2003) Maximum tolerable dose and low-dose metronomic chemotherapy have opposite effects on the mobilization and viability of circulating endothelial progenitor cells. Cancer Res 63(15):4342–4346 12907602

[183] Shaked Y, Bertolini F, Man S et al (2005) Genetic heterogeneity of the vasculogenic phenotype parallels angiogenesis; Implications for cellular surrogate marker analysis of antiangiogenesis. Cancer Cell 7(1):101–111 15652753 10.1016/j.ccr.2004.11.023

